# Structure of Microtubule-Trapped Human Kinesin-5 and Its Mechanism of Inhibition Revealed Using Cryoelectron Microscopy

**DOI:** 10.1016/j.str.2020.06.003

**Published:** 2021-07-01

**Authors:** Alejandro Peña, Aaron Sweeney, Alexander D. Cook, Julia Locke, Maya Topf, Carolyn A. Moores

## Main text

(Structure *28*, 450–457.e1–e5; April 7, 2020)

In the originally published version of this article, Dr. Julia Locke was mistakenly omitted from the author list. The corrected author list appears here and has been corrected online, and the Author Contributions and Acknowledgments have been updated accordingly.

The authors regret this error.

