# Supplementary material for: Structure of Microtubule-Trapped Human Kinesin-5 and Its Mechanism of Inhibition Revealed Using Cryoelectron Microscopy
Source: Structure. 2020 Apr 7;28(4):450–457.e5. doi: 10.1016/j.str.2020.01.013 (PMC7139217; doi:10.1016/j.str.2020.01.013)
Supplement: Document S1. Figures S1–S3 [file mmc1.pdf]

Structure, Volume *28*

Supplemental Information

Structure of Microtubule-Trapped Human Kinesin-5 and Its Mechanism of Inhibition Revealed Using Cryoelectron Microscopy

Alejandro Peña, Aaron Sweeney, Alexander D. Cook, Maya Topf, and Carolyn A. Moores

Structure of microtubule-trapped human kinesin-5 and its mechanism of inhibition revealed using cryo-EM

Alejandro Peña [1,2], Aaron Sweeney [1], Alexander D. Cook [1], Maya Topf [1] and Carolyn A. Moores [1]*

[1] Institute of Structural and Molecular Biology, Birkbeck College, London WC1E 7HX, U.K.
[2] Current address: Department of Life Sciences, Medical Research Council Centre for Molecular Bacteriology & Infection, Imperial College London, London SW7 2BX, UK

* Corresponding Author/Lead Contact
Carolyn A. Moores; ORCID ID: 0000-0001-5686-6290
E-mail: c.moores@mail.cryst.bbk.ac.uk

Supplemental Figures

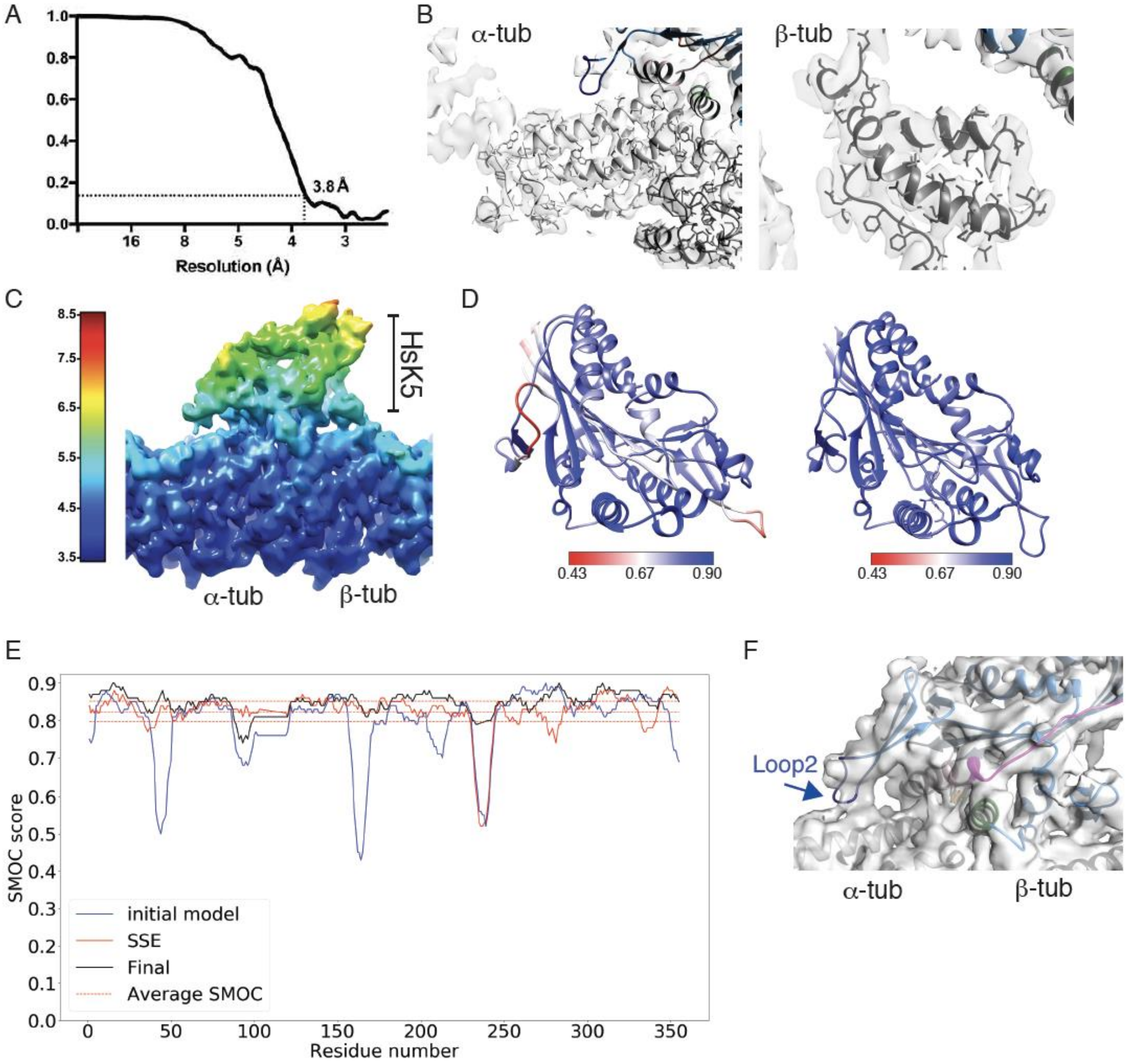

Figure S1 Related to Figure 2. Evaluation of the MT-trapped HsK5 bound by GSK-1 cryo-EM reconstruction. (A) FSC curve for the overall reconstruction estimated to be 3.8 Å by the 0.143 criterion; (B) Visualization of near-atomic resolution features in the MT regions of the density supports the near-atomic resolution estimate of the reconstruction; (C) Depiction of the local resolution estimate in the reconstruction using RELION (Zivanov et al., 2018) indicates the presence of a resolution gradient between the MT and the kinesin; (D) The calculated HsK5 atomic model before (left) and after (right) Flex-EM refinement in the MT-bound HsK5-GSK-1 cryo-EM density were assessed with the TEMPy SMOC score (Joseph et al., 2016) used here to color the final coordinates, illustrating the high quality of the fit; (E) Plot of SMOC scores calculated sequentially for the initial model, after refinement of secondary structural elements (SSEs) and for the final model; (F) At inclusive density thresholds, connectivity is seen between HsK5 loop2 and α-tubulin (arrow), although this more inclusive density was not included as part of the modelling procedure.

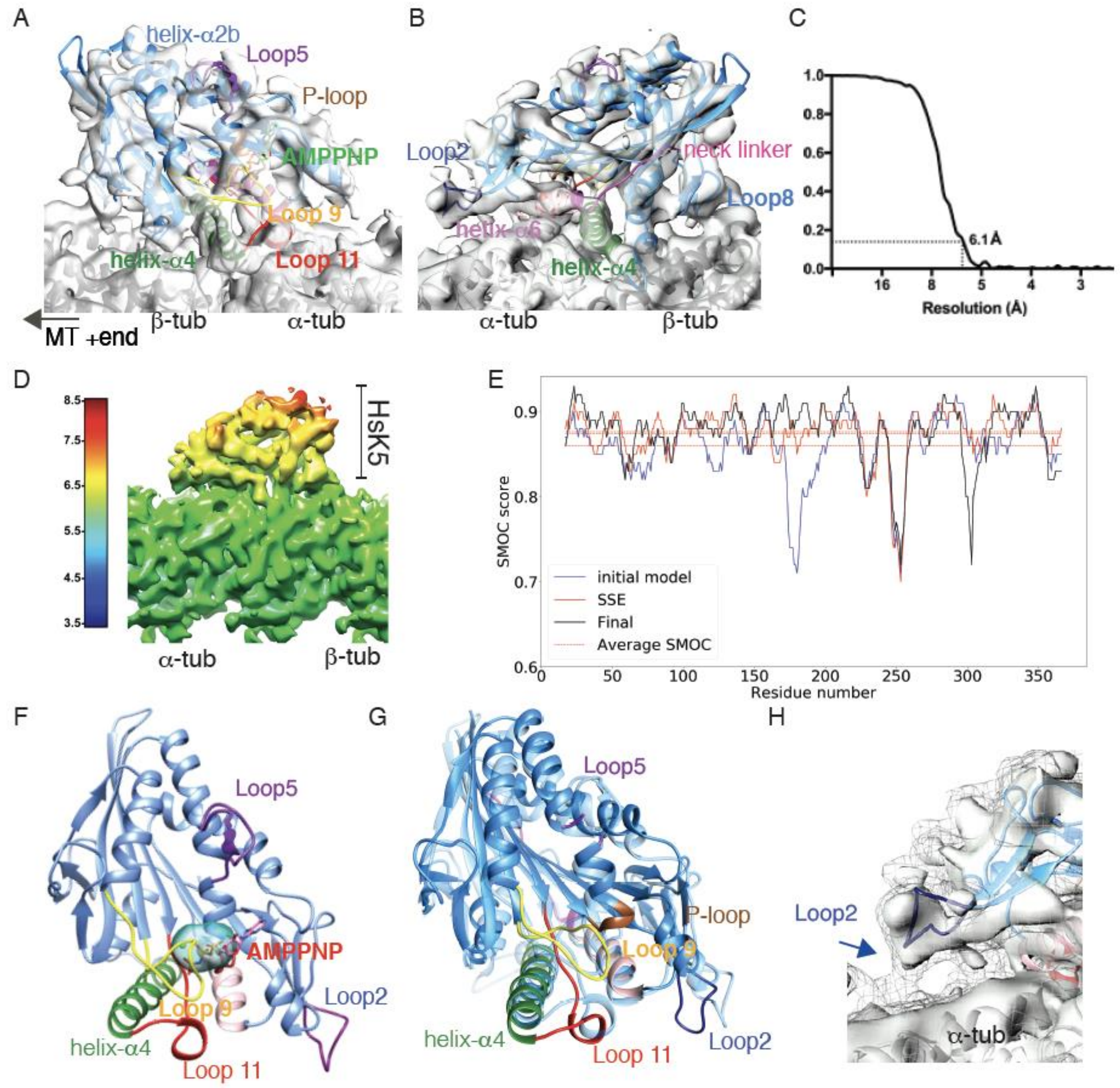

Figure S2 Related to Figure 2. Evaluation of the MT-bound HsK5-AMPPNP cryo-EM reconstruction resolution. (A) An asymmetric unit from the HsK5-AMPPNP-MT reconstruction contoured to show secondary structural elements, and viewed with the MT plus end on the left; the cryo-EM densities are shown as a grey surface representation, α- and β-tubulin are shown in light and dark grey ribbons respectively and the HsK5 model is shown in coloured ribbons with individual secondary structural elements highlighted (helix-α4, green; P-loop, brown; switch 1, loop 9, yellow; switch 2, loop 11, red; loop5, purple; bound nucleotide (modelled as AMPPNP, based on prior knowledge (Goulet et al., 2014) and consistent with sample preparation conditions but not directly identified as such from the density) is shown in stick representation); (B) An asymmetric unit from the HsK5-AMPPNP-MT reconstruction contoured as in (A) viewed with the MT plus end on the right (helix-α6, pink; loop2, dark blue; neck-linker, fuchsia); (C) FSC curve for the overall reconstruction estimated to be 6.1 Å by the 0.143 criterion; (D) Depiction of the local resolution estimate in the reconstruction using RELION (Zivanov et al., 2018); (E) Plot of SMOC scores calculated sequentially for the initial model, after refinement of SSEs and for the final model; note the different scale compared to Figure S1E for ease of visualization; (F) Calculated map vs protein model difference (in turquoise) identifies density corresponding to bound nucleotide

(particularly the phosphate moieties, here modelled as AMPPNP) at the HsK5 nucleotide binding site; the AMPPNP-bound HsK5 conformation is shown in blue ribbons with individual secondary structural elements highlighted as in (A); (G) Overlay of the models of MT-bound HsK5 bound to GSK-1 or AMPPNP, viewed as in panel A and at 90º compared to Figure 2B; the GSK-1-bound conformation of HsK5 is shown in mid-blue/coloured ribbon, while the AMPPNP-bound HsK5 conformation is shown in light blue; GSK-1/AMPPNP have been removed for clarity; (H) At inclusive density thresholds, connectivity between loop2 and α-tubulin is seen in HsK5-AMPPNP reconstruction (arrow), although it was not possible to model the structure of this loop, consistent with the flexibility of this connection.

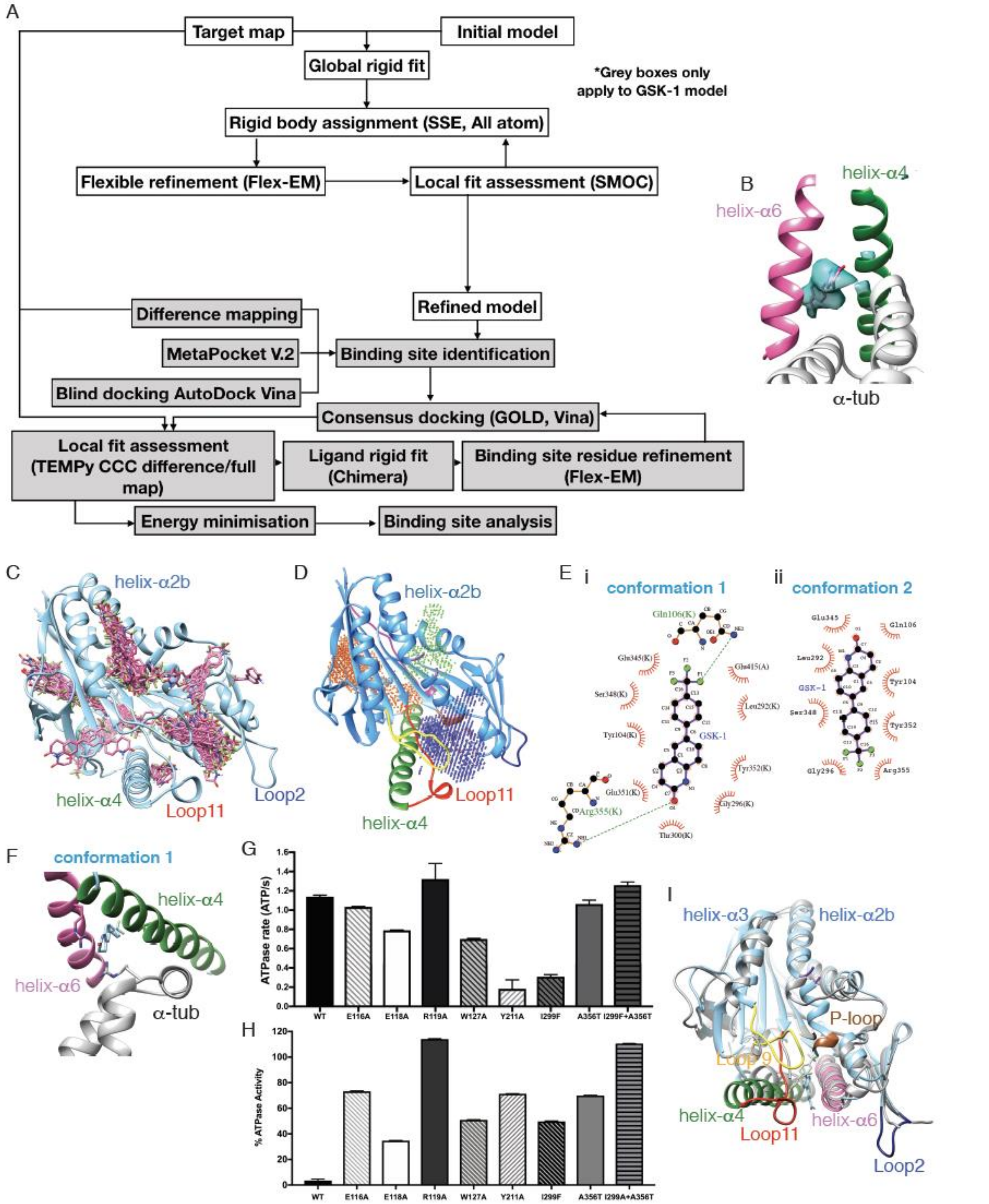

Figure S3 Related to Figure 3. Evaluation of the GSK-1 binding site to MT-bound HsK5. (A) Protocol for computationally identifying and characterising the GSK-1 binding site on MT-bound GSK-1; (B) Calculated map vs protein model difference (in turquoise) identifies major density corresponding to bound GSK-1, in the helix-α4/helix-α6; (C) Results of blind docking with AutoDock Vina (after removal of redundant poses < 2 Å) in the absence of MTs. The refined HsK5 model is shown in blue and unique GSK-1 conformations are in pink. Several discrete clusters of GSK-1 binding can be seen - their distribution is less clustered at the helix-α4/helix-α6 pocket in the absence of tubulin compared to the presence (Figure 3B); (D)

The top 3 clusters predicted by Meta-pocket 2.0 (purple, green, and, orange) shown in relation to the refined GSK-1 bound HsK5 model (blue). SSEs are also highlighted (helix-α4, green; P-loop, brown; loop11, red); (E) 2D representations generated LigPlot of the drug pocket for GSK-1 (i) conformation 1 and (ii) conformation 2; individual atoms and interactions residues are shown (C: black; O: red; N:blue; F:green); binding site residues that interact with GSK-1 via hydrophobic interactions (red semi-circles) are also indicated; (F) Depiction of the contribution of α-tubulin-Arg402 to GSK-1 binding by HsK5 as predicted by PLIP; (G) Effect of point mutagenesis on uninhibited HsK5 ATPase activity - bar graphs show mathematical mean with S.D. indicated by bars, n= 3 or 4 for each condition; (H) Sensitivity of the MT-stimulated ATPase activity of these HsK5 mutants to inhibition by 50 nM GSK-1, expressed as a % of the uninhibited activity, n= 3 or 4 for each condition; (I) Overlay of our HsK5-GSK-1-MT structure (colored as in previous figures, but depicted without tubulin) with the PVZB119-bound HsK5 structure (PDB: 3WPN, in grey (Yokoyama et al., 2015), highlighting the similarity of the drug binding sites (shown in stick representation) but also showing the extent of secondary structure disorder in HsK5-PVZB119 around the nucleotide binding site in the absence of MT binding.
