# Supplementary material for: Structure of Microtubule-Trapped Human Kinesin-5 and Its Mechanism of Inhibition Revealed Using Cryoelectron Microscopy
Source: Structure. 2020 Apr 7;28(4):450–457.e5. doi: 10.1016/j.str.2020.01.013 (PMC7139217; doi:10.1016/j.str.2020.01.013)
Supplement: Document S2. Article plus Supplemental Information [file mmc2.pdf]

Short Article

Structure

Structure of Microtubule-Trapped Human Kinesin-5 and Its Mechanism of Inhibition Revealed Using Cryoelectron Microscopy

Graphical Abstract

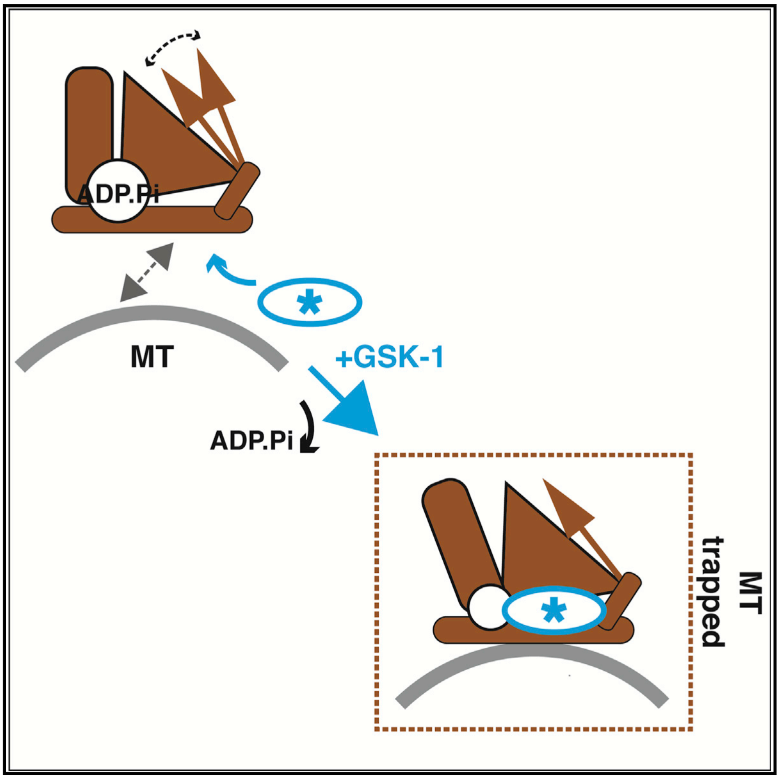

Highlights

- GSK-1 inhibits human kinesin-5 monomers thereby promoting microtubule stabilization
- GSK-1 binds between helix-α4 and helix-α6 at the motor-microtubule interface
- GSK-1 traps the kinesin-5 microtubule binding interface in an ATP-like state
- GSK-1 perturbs the nucleotide binding site thereby preventing ATP binding

Authors

Alejandro Peña, Aaron Sweeney, Alexander D. Cook, Maya Topf, Carolyn A. Moores

Correspondence

c.moores@mail.cryst.bbk.ac.uk

In Brief

To investigate mitotic kinesin inhibition mechanisms, we used cryo-EM to determine the 3.8-Å resolution structure of human kinesin-5 trapped on the microtubule by the drug GSK-1. GSK-1 binds between helix-α4 and helix-α6 in the motor domain, stabilizing the motor's microtubule binding interface while allosterically preventing nucleotide binding.

Peña et al., 2020, Structure *28*, 450–457
April 7, 2020 © 2020 The Author(s). Published by Elsevier Ltd.
https://doi.org/10.1016/j.str.2020.01.013

Structure
Short Article

Structure of Microtubule-Trapped Human Kinesin-5 and Its Mechanism of Inhibition Revealed Using Cryoelectron Microscopy

Alejandro Peña,[1,2] Aaron Sweeney,[1] Alexander D. Cook,[1] Maya Topf,[1] and Carolyn A. Moores[1,3,*]
[1]Institute of Structural and Molecular Biology, Birkbeck College, London WC1E 7HX, UK
[2]Present address: Department of Life Sciences, Medical Research Council Centre for Molecular Bacteriology & Infection, Imperial College London, London SW7 2BX, UK
[3]Lead Contact
*Correspondence: c.moores@mail.cryst.bbk.ac.uk
https://doi.org/10.1016/j.str.2020.01.013

SUMMARY

Kinesin-5 motors are vital mitotic spindle components, and disruption of their function perturbs cell division. We investigated the molecular mechanism of the human kinesin-5 inhibitor GSK-1, which allosterically promotes tight microtubule binding. GSK-1 inhibits monomeric human kinesin-5 ATPase and microtubule gliding activities, and promotes the motor's microtubule stabilization activity. Using cryoelectron microscopy, we determined the 3D structure of the microtubule-bound motor-GSK-1 at 3.8 Å overall resolution. The structure reveals that GSK-1 stabilizes the microtubule binding surface of the motor in an ATP-like conformation, while destabilizing regions of the motor around the empty nucleotide binding pocket. Density corresponding to GSK-1 is located between helix-α4 and helix-α6 in the motor domain at its interface with the microtubule. Using a combination of difference mapping and protein-ligand docking, we characterized the kinesin-5-GSK-1 interaction and further validated this binding site using mutagenesis. This work opens up new avenues of investigation of kinesin inhibition and spindle perturbation.

INTRODUCTION

Kinesins are ATP-dependent motors that move along microtubules (MTs), organize them, and modify their dynamics. Despite being defined by their conserved motor domains, sequence variations within individual kinesin families enable them to perform specialized functions. Kinesins are important components of the mitotic spindle, and their activities are carefully coordinated to ensure accurate distribution of replicated DNA to daughter cells. Kinesin-5s are important for the assembly and maintenance of spindle bipolarity. They are dumbbell-shaped tetramers with pairs of motor domains at either end (Goulet and Moores, 2013). This molecular layout enables them to crosslink and slide MTs by moving toward their plus ends. Thus, the dynamic interaction of kinesin-5s with spindle MTs generates force that pushes and holds spindle poles apart (Mann and Wadsworth, 2019). As well as MT-based motility, kinesin-5s can also promote MT polymerization (Chen et al., 2019; Chen and Hancock, 2015). The molecular mechanisms by which kinesin-5s couple their ATPase activity to nucleotide-dependent conformational changes that drive motility and MT sliding are increasingly well understood (Goulet and Moores, 2013; Mann and Wadsworth, 2019).

Human kinesin-5 (also known as Eg5, kinesin spindle protein, KIF11) was the first mitotic kinesin for which specific small-molecule inhibitors were identified (Mayer et al., 1999). These inhibitors are valuable tools in dissecting both the role of this motor within the complex spindle machinery and in investigating its mechanochemistry. Because the mitotic spindle has long been considered a major target of anticancer therapies, the discovery of small molecules specific to mitotic kinesins also sparked major interest in these motors as anticancer targets (Rath and Kozielski, 2012). Although there are open questions about the usefulness of anti-mitotics in cancer therapy (Komlodi-Pasztor et al., 2013), perturbation of cell division—e.g., via disruption of mitotic kinesin function—may reduce tumor fitness and/or stimulate an immune response. Anti-mitotics thus continue to be investigated in the context of cancer treatment (Chandrasekaran et al., 2015; Funk et al., 2016; Mitchison, 2012).

Human kinesin-5 inhibitors are chemically diverse (Good et al., 2011), but nearly all of them act by preventing tight MT binding by the motor: drug-bound kinesin-5 is trapped in its ADP state, which has low MT affinity, thereby blocking force generation within the spindle (Crevel et al., 2004; Kwok et al., 2006; Mayer et al., 1999). These inhibitors bind at the same allosteric site in the motor domain, between helix-α2 and -α3 and enclosed by loop5 (Yan et al., 2004). The kinesin-5-specific sequence and structure of this binding site explains inhibitor specificity, and their mode of action explains why they cause mitotic spindle collapse. It also explains why cells cultured in the presence of such kinesin-5 inhibitors can evolve to allow an alternative mitotic kinesin—kinesin-12—to take over force generation activity within the spindle (Sturgill et al., 2016).

In contrast to these loop5-binding small molecules, several other kinesin-5 inhibitors have been described as promoting and specifically inhibiting the MT-bound state in human kinesin-5 (Chattopadhyay et al., 2015; Chen et al., 2017; Groen

 © 2020 The Author(s). Published by Elsevier Ltd.
This is an open access article under the CC BY license (http://creativecommons.org/licenses/by/4.0/).

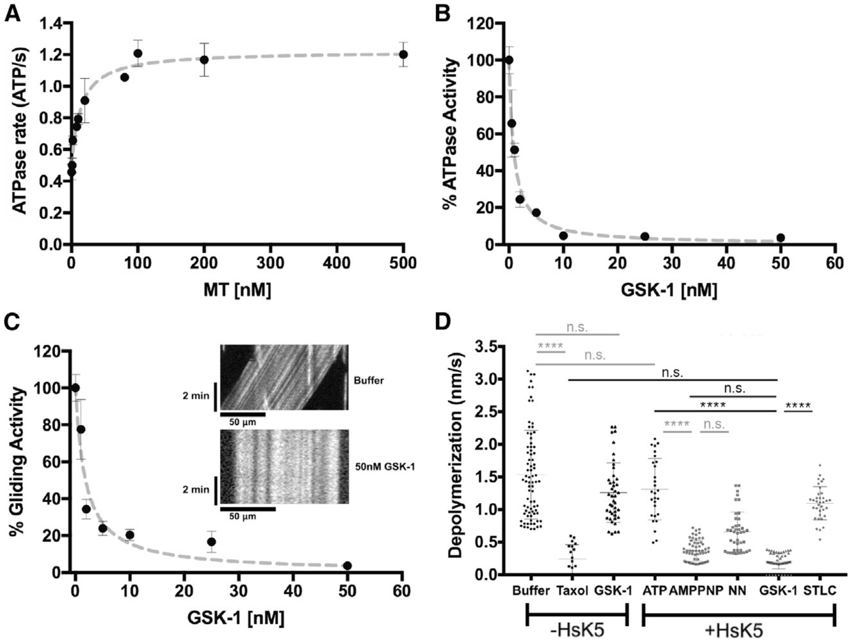

Figure 1. GSK-1 Induces Tight MT-Bound Inhibition of Human Kinesin-5, Blocking the Motor ATPase and MT Gliding Activity, and Stabilizing MT End

(A) HsK5 steady-state ATPase rate plotted as a function of [MT]. Data were fit to a Michaelis-Menten kinetic using GraphPad Prism yielding values for V_{max} = 1.22 ± 0.07 ATP/s and $K_{0.5}MT$ = 13.5 ± 3.3 nM. Error bars represent the mathematical mean ± SD for each MT concentration, n = between 4 and 12 for each MT concentration. (B) Inhibition of HsK5 ATPase activity by GSK-1 titration, with an IC_{50} of 0.8 nM; the mathematical mean and SD are plotted for each condition, n = between 4 and 7 for each MT concentration, and the curve was fitted using GraphPad Prism; R^2 = 0.998. (C) Inhibition of HsK5 gliding activity by GSK-1 titration, with an IC_{50} of 1.8 nM; the mathematical mean and SD are plotted for each condition (for the GSK-1 titration, n = 129, 27, 82, 80, 47, 11, and 58) and the curve was fitted using GraphPad Prism; R^2 = 0.937. Inset, exemplar kymographs demonstrating HsK5-driven MT gliding velocity and its inhibition by GSK-1 (following + ATP buffer washes). (D) Plot of the rate of depolymerization of paclitaxel-stabilized MTs measured using total internal reflection fluorescence microscopy following washes in the absence/presence of various ligands, including HsK5—scatterplot of all the data with mean and SD indicated by horizontal bars; for –HsK5 buffer control, n = 79, +Taxol (20 μM), n = 19, +GSK-1 (50 nM, n = 43; for + HsK5 +ATP, n = 28, +HsK5 +AMPPNP, n = 58, +HsK5 NN (no nucleotide), n = 42, +HsK5 +GSK-1, n = 45, +HsK5 +STLC (50 nM S-trityl-L-cysteine), n = 35. A one-way ANOVA was performed on all these data in Prism to establish the significance of the nucleotide-dependent differences, with those directly relating to the effect of GSK-1 binding to HsK5 shown in black. ****p < 0.0001; n.s., not significant (p > 0.01).

et al., 2008; Luo et al., 2007, 2008; Tarui et al., 2014). This mode of inhibition—which has also been characterized in several other mitotic kinesins (Dumas et al., 2019; Locke et al., 2017; Wu et al., 2013)—offers a different perspective on kinesin activity within the spindle and provides an alternative way to perturb spindle function.

The structural basis of human kinesin-5 inhibition by small molecules that promote tight MT binding is not understood. To shed light on this, we investigated inhibition of human kinesin-5 by the biaryl compound GSK-1 (Luo et al., 2007). GSK-1 is an ATP-competitive, MT-uncompetitive allosteric inhibitor with a K_i in the low nM range (Luo et al., 2007). It specifically inhibits MT-stimulated—and not basal—kinesin-5 ATPase activity, and its binding site was previously modeled at the junction of helices-α4 and -α6 in the kinesin-5 motor domain (Luo et al., 2007). Using a monomeric human kinesin-5 motor domain construct (HsK5), we measured GSK-1 inhibition of MT-stimulated ATPase and multi-motor gliding activity, confirming that GSK-1 induces tight binding of the motor to its MT track, and also promotes stabilization of MTs against depolymerization. We imaged the HsK5-MT-GSK-1 complex using cryoelectron microscopy (cryo-EM), calculated its 3D structure to an overall resolution of 3.8 Å, and used this to build an atomic model of the complex. This structure reveals the dramatic consequences of GSK-1 binding on the conformation of HsK5, stabilizing its MT binding surface and destabilizing regions of the motor domain adjacent to the empty nucleotide binding pocket. We also identified the allosteric binding site of GSK-1 on the MT-bound kinesin-5, between helix-α4 and -α6 at the interface with the MT, consistent with previous modeling (Luo et al., 2007), and validated this site of inhibition using mutagenesis. This work provides a structural explanation for how such inhibitors trap human kinesin-5 on MTs, opening up new avenues of investigation of kinesin inhibition and mitotic spindle perturbation.

RESULTS AND DISCUSSION

GSK-1 Inhibition of Human Kinesin-5 Promotes Tight MT Binding

The MT-stimulated steady-state ATPase activity of our monomeric human kinesin-5 construct (HsK5, V_{max} = 1.22 ± 0.07 ATP/s, $K_{0.5}$ MT = 13.5 ± 3.3 nM) (Figure 1A) is consistent with previous reports (e.g., Cochran et al., 2004). Our HsK5 construct was inhibited by GSK-1 with a half maximal inhibitory concentration (IC_{50}) of 0.8 nM (Figure 1B), also consistent with previous reports (Luo et al., 2007). Similarly, in a multi-motor MT gliding assay, where HsK5 exhibited an average uninhibited gliding velocity of 26 nm/s, HsK5 activity was inhibited by GSK-1 with an IC_{50} of 1.8 nM (Figure 1C). Even after multiple washes with buffer, GSK-1 caused the MTs to remain stationary and tightly attached in the assay chamber (Figure 1C, inset), consistent with GSK-1 acting by stabilizing the HsK5-MT complex (Tarui et al., 2014). This could be because of the high affinity of drug binding, but could also be because, sterically, the drug is not readily released from its binding site in the HsK5-MT complex.

As well as MT gliding activity, human kinesin-5 has also been shown to stabilize MT ends, favoring their growth (Chen et al., 2019; Chen and Hancock, 2015). We therefore investigated the

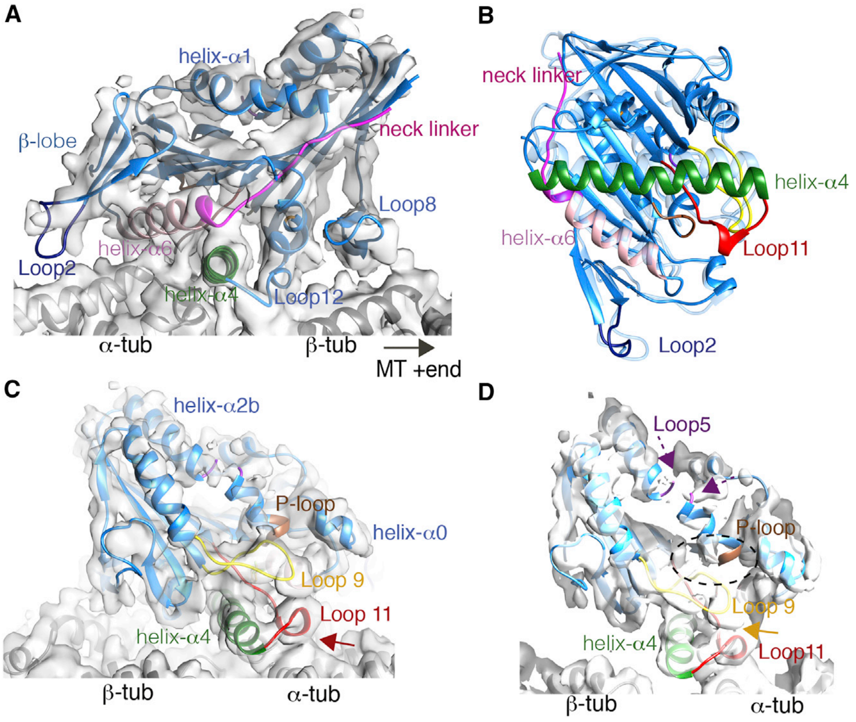

Figure 2. Cryo-EM Structure of MT-Bound HsK5 in the Presence of GSK-1

(A) An asymmetric unit from the HsK5-GSK-1-MT reconstruction contoured to show secondary structural elements, and viewed with the MT plus end on the right. The cryo-EM densities are shown as a gray surface representation, α- and β-tubulin are shown in light and dark gray ribbons, respectively, and the HsK5 model is shown in colored ribbons with individual secondary structural elements highlighted (helix-α4, green; helix-α6, pink; loop2, dark blue; neck linker, fuchsia).

(B) MT binding interface viewed from the MT toward HsK5-GSK-1 (blue and colored ribbon), overlaid using tubulin for alignment on the MT-bound HsK5-AMPPNP model (light blue ribbon) revealing the similarity of this interface which supports neck linker docking.

(C) An asymmetric unit from the HsK5-GSK-1-MT reconstruction contoured to show secondary structural elements, viewed with the MT plus end on the left; helix-α4, green; P loop, brown, switch I/loop9, yellow, switch II/loop11, red; red arrow indicates limited contact between loop11 and α-tubulin.

(D) Views toward the empty nucleotide binding site, with conserved nucleotide binding loops colored (P loop, brown, switch I/loop9, yellow, switch II/loop11, red); the position where density corresponding to nucleotide would be but is allosterically prevented by GSK-1, is indicated with a dotted circle; orange arrow indicates contact between loop9 and loop11, dotted purple arrows indicate absence of density for loop5. See also Figures S1 and S2.

effect of our monomeric construct in different biochemical states on MT stability. When paclitaxel-stabilized MTs are washed with buffer in the absence of paclitaxel, MT ends slowly depolymerize (1.50 ± 0.07 nm/s), an effect that is suppressed by the addition of paclitaxel to the wash (0.24 ± 0.05 nm/s; Figure 1D). Inclusion of HsK5 in different biochemical states in the wash step resulted in a range of stabilization effects—the fold increase in stability compared with no paclitaxel are: +ATP = 1.2-fold; no added nucleotide = 2.3-fold; +AMPPNP = 3.8-fold, consistent with the reported affinity of HsK5 for the MT in each condition (Chen et al., 2017). In the presence of HsK5+GSK-1, stabilization equivalent to that in the presence of paclitaxel (8-fold increase in stability) was observed, while addition of GSK-1 in the absence of HsK5 had no effect on MT stability (Figure 1D). Thus, the tight MT binding promoted by GSK-1 also enhances MT end stabilization by HsK5.

The Structure of MT-Trapped GSK-1 Bound HsK5

Since GSK-1 acts by trapping the HsK5 motor on the MT, we used cryo-EM to image the HsK5-MT complex in the presence of GSK-1, determined its structure to an overall resolution of 3.8 Å (with resolution in the kinesin motor domain between ~4.5 and 8 Å, Figures S1A–S1C), and calculated an atomic model for the complex (Figures 2A, S1D, and S1E). Density corresponding to the MT binding surface of HsK5—encompassing loop8, helix-α4, loop12, helices-α5 and -α6—is very well defined. Comparison with available HsK5 structures (in particular PDB: 3HQD) and with our improved cryo-EM reconstruction of MT-bound HsK5-AMPPNP (Figures S2A–S2E), showed that this portion of HsK5 adopts an ATP-like configuration in the presence of GSK-1 (Figures 2B and S2F). Consistent with this, density corresponding to the neck linker aligns along the edge of the motor domain central β sheet, directed toward the MT plus end (Figure 2A). The β sheet1 lobe that lies on top of helix-α6 is also well defined (Figure 2A). Loop2 extends from this region and, at low density thresholds, connects to the surface of the underlying α-tubulin (Figure S1F). This additional point of contact with the MT surface, together with the previously described role for loop11-helix-α4 (Chen et al., 2019), could allow HsK5 to influence the conformation of the underlying tubulin and thereby explain the motor's MT end stabilizing activity; this is particularly pronounced in the presence of GSK-1 but may act in some other nucleotide states, albeit more dynamically (Figure S2G). On the opposite side of HsK5 and adjacent to the nucleotide binding site, the characteristic helical turn in loop11 makes minimal contact with the surface of α-tubulin (Figure 2C, red arrow). Density connects loop11 with the adjacent loop9, a configuration also associated with ATP-like state of the motor (Figure 2D, orange arrow).

Strikingly, however, the nucleotide binding site itself is empty in the HsK5-GSK-1 structure, and while density corresponding to switch I in loop9 is clear, only discontinuous density is seen around the nucleotide binding site including very little density corresponding to the P loop being visible (Figure 2D, dotted oval). Above the nucleotide binding site, the cryo-EM density on the outer surface of the central β sheet facing away from the MT surface is less clearly defined (Figure 2D). A resolution gradient is characteristic of all kinesin-MT reconstructions to

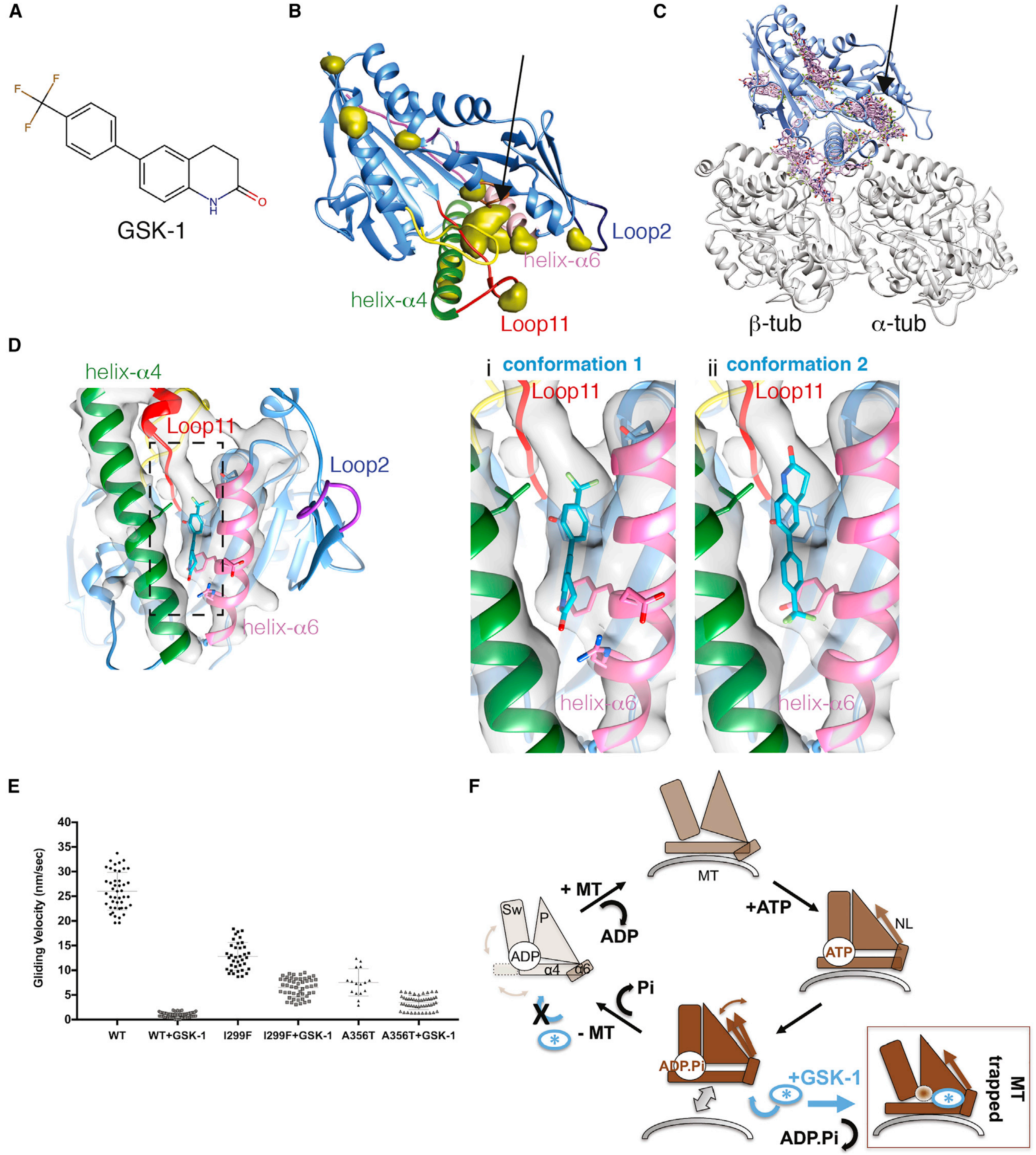

Figure 3. Identification and Validation of the GSK-1 Binding Site on MT-Bound HsK5

(A) Depiction of GSK-1.

(B) Positive difference density (in yellow) calculated by the subtraction of the HsK5-AMPPNP reconstruction from the HsK5-GSK-1 reconstruction. The positions of difference density are shown relative to the HsK5 ribbon model, with parts of HsK5 colored as previously (helix-α4, green; helix-α6, pink; loop2, dark blue; loop11, red). Density that could not be accounted for by the models is highlighted (black arrow) within the pocket between helix-α4 and helix-α6.

(C) The results of blind docking with AutoDock Vina (after removal of redundant poses <2 Å) in the presence of MTs. The refined HsK5 model is shown in blue, α- and β-tubulin are shown in gray, and unique GSK-1 conformations are in pink. Several discrete clusters of GSK-1 binding are observed.

(legend continued on next page)

date, due in part to greater deviation from pseudo-helical symmetry at higher MT radii, and potentially also to some conformational flexibility in the motor domain itself. Such a (relatively gentle) gradient is seen in our HsK5-AMPPNP (Figure S2). However, the resolution gradient is more marked in the HsK5-GSK-1 reconstruction, which itself has a much higher resolution overall (Figure S1C). Density corresponding to helices-α1, -α2, and -α3 is less well defined compared with the MT binding region, which indicates that there is substantial conformational structural variation in this region of the motor domain in the presence of GSK-1. Furthermore, there is minimal density corresponding to loop5 (Figure 2D).

The ATP-like conformation of the MT-contacting regions within HsK5 in this reconstruction helps to explain the tight association of the motor in the presence of GSK-1. GSK-1 has been shown kinetically to be ATP-competitive, but the nucleotide binding site in our reconstruction is empty, supporting the indirect nature of GSK-1's competition with ATP (Luo et al., 2007). The disorder in key nucleotide binding motifs are therefore likely to be the structural consequence of a lack of bound nucleotide in this structure. Overall, the presence of GSK-1 enables us to visualize MT-trapped HsK5 in a previously uncharacterised conformation.

GSK-1 Binding Site and Mechanism of Inhibition

To identify the GSK-1 (Figure 3A) binding site itself, as described previously (Locke et al., 2017), we first used TEMPy (Farabella et al., 2015) to calculate the difference density between the HsK5-GSK-1 and HsK5-AMPPNP cryo-EM reconstructions (Figures 3B and S3A). Some of the difference peaks correspond to regions of conformational difference within the HsK5 motor domain itself in the two states, for example, within loop2 and loop11 (Figure S2F). However, there was also a conspicuous peak between helix-α4 and -α6, adjacent to the motor-MT interface where the quality of the HsK5 cryo-EM density is particularly good (Figure 3B, arrow). This density was not accounted for by the HsK5 model (Figure S3B) and is consistent with the previously identified binding region of GSK-1 using peptide sequencing (Luo et al., 2007). Furthermore, this region is where other biaryl compounds have been reported to interact with HsK5 (Ulaganathan et al., 2013; Yokoyama et al., 2015).

To further investigate the drug-binding properties of this pocket, binding site prediction was used in combination with blind docking (Figures S3A, S3C, and S3D, see the STAR Methods). Blind docking identified a range of putative binding sites (Figure S3C), including the site between helix-α2 and -α3 adjacent to loop5, where monastrol-type HsK5 inhibitors bind, as well as in the pocket between helix-α4 and -α6 (Figure 3C). The binding site prediction also showed that the region of HsK5 between helix-α4 and -α6 was suitable for GSK-1 binding, while the additional potential docking sites identified elsewhere on HsK5 did not coincide with the calculated difference densities and/or clashed with regions of previously modeled protein structure (Figure S3D). Together, with the difference density data, we therefore concluded based on our data that GSK-1 indeed binds in the pocket between helix-α4 and -α6 in MT-bound HsK5.

Next, using the docking prediction information in combination with the difference density, we applied a two-stage consensus docking protocol (Figure S3A), and identified 17 conformations for GSK-1 between helix-α4 and -α6. These conformations were analyzed individually and two alternative conformations—one each from AutoDock Vina and from GOLD Chemscore—were found to fit equally well (Figure 3D), with cross-correlation coefficients of 0.62 (conformation 1) and 0.57 (conformation 2) with the difference map density and 0.82 (1) and 0.80 (2) with the full map density. The quality of the cryo-EM density in this region of the reconstruction is only sufficient to identify the overall location of the ligand, and is not of sufficiently high resolution to distinguish between these predicted conformations. However, the computational docking approaches described in the previous paragraph provide validation of these poses: they are essentially rotated by approximately 180° with respect to each other in the binding site—the trifluoromethyl moiety is directed toward the P loop in conformation 1 (Figure 3Di) and toward the junction of helix-α4 and -α6 in conformation 2 (Figure 3Dii). Conformation 1 is most similar to previous predictions of GSK-1 binding (Luo et al., 2007), although our structure suggests that GSK-1 is positioned further toward the P loop in the MT-bound state of HsK5. In both predicted conformations, the aromatic rings of GSK-1 are well-accommodated within the relatively hydrophobic environment of this part of HsK5 (Figure S3E). Interestingly, PLIP (Salentin et al., 2015) predicted an additional interaction between GSK-1 in conformation 1 and α-tubulin-Arg402, which has been previously indicated in motor-tubulin interactions (Figure S3F) (Cao et al., 2014; Uchimura et al., 2015). The contribution of a tubulin residue to GSK-1 binding could help to explain this drug's specificity for tubulin-bound HsK5.

To investigate this GSK-1 binding site further, we mutated previously investigated HsK5 residues both at the identified site between helix-α4 and -α6 (Luo et al., 2007), and at the loop5 allosteric site. We verified the activity of these mutants and, as was previously observed in other kinesins (e.g, Locke et al., 2017), some of these mutations influence the uninhibited activity of the motor (Figure S3G). Nevertheless, whereas

(D) Left, view of the cryo-EM density toward the GSK-1 binding pocket (black dashed rectangle); right, zoomed in views showing the predicted (i) conformation 1 and (ii) conformation 2 of GSK-1; side chains of Gln106 and Arg355, predicted to be involved in GSK-1 binding in conformation 1 (Figure S3D), are depicted in stick representation.

(E) Sensitivity of gliding activity of HsK5 point mutants to inhibition by 50 nM GSK-1; scatterplot of all the data with mean and SD indicated by horizontal bars; data for wild-type (WT) are the same as used in Figure 1C; for HsK5 WT, n = 43, HsK5 WT ± GSK-1 n = 18; I229F n = 38, I229F + GSK-1 n = 47; A356T n = 14, A356T + GSK-1 n = 48; a pairwise t test was performed in Prism to establish the significance of these mutants compared with the WT and ± GSK-1, all of which showed significant statistical difference ($p < 0.0001$). See also Figure S3.

(F) Schematic of the HsK5 MT-dependent ATPase cycle, in which the subdomains within the motor domain (SwI/II, Sw; P loop, P; tubulin-binding, represented helix-α4, α4), together with helix-α6 (α6) and the neck linker (NL) reorganize in response to MT binding and bound nucleotide (Goulet et al., 2014); we suggest that the GSK-1 binding site is accessible and conformationally favored as HsK5 releases from the MT in the ADP.Pi state; this then re-traps HsK5-GSK-1 on the MT with the subdomains in a non-physiological configuration in which the P loop region is distorted such that nucleotide cannot bind.

50 nM GSK-1 completely inhibits wild-type HsK5, I299F, and A356T each maintain 50%–60% of their uninhibited activity in both ATPase and MT gliding assays in the presence of 50 nM GSK-1 (Figures 3E and S3H). These mutants are thus less sensitive to GSK-1 inhibition, likely because the substitution of larger side chains reduces drug binding at that site. Interestingly and as was also observed previously (Luo et al., 2007), some of the loop5 mutants also exhibited increased resistance to GSK-1 (Figure S3H), which our structure can now explain due to the allosteric effects of GSK-1 binding in the vicinity of the nucleotide binding site.

Here, our data show that GSK-1 traps HsK5 on MTs by binding between helix-α4 and -α6. Kinesin motor domains are composed of three distinct subdomains—tubulin-binding, switch I/II, and P loop subdomains (Figure 3F)—which move with respect to each other during the motor's MT-bound ATPase cycle (Cao et al., 2014; Shang et al., 2014). The GSK-1 binding site lies at the junction of the tubulin-binding and P loop subdomains and induces a tight MT binding state of the motor. Simultaneously, GSK-1 prevents nucleotide binding through perturbation of the P loop, and thereby causes significant structural distortion of regions adjacent to the empty nucleotide binding site (Figure 3F).

The HsK5 inhibitor PVZB1194 is also a biphenyl compound, ATP-competitive, and specific for MT-stimulated HsK5 ATPase (Yokoyama et al., 2015). PVZB1194 also binds at the junction of helix-α4 and -α6, and the HsK5-PVZB1194 X-ray structure together with our HsK5-GSK-1-MT present a consistent picture of the multiple allosteric structural consequences of HsK5 inhibition by biaryl inhibitors at this site (Figure S3I). However, much of the PVZB119-bound HsK5 structure—the N-terminal half of helix-α4, loop9, loop11, and loop5—is unusually flexible and therefore not visualised. Because both GSK-1 and PVZB1194 specifically inhibit MT-bound HsK5, the structure we have determined seems more likely to be the bona fide inhibitory complex. The configuration of HsK5 that we observed is probably rarely sampled in the absence of MTs—which in particular stabilizes and constrains the elongated state of helix-α4—and is thus consistent with the absence of inhibition of GSK-1 on basal HsK5 ATPase activity (Luo et al., 2007). Although our cryo-EM data are not of sufficient resolution to unambiguously describe the configuration of the bound GSK-1, the shape of the binding site in MT-bound HsK5 is clear; this suggests that, although distinct from previous structures and predictions, our structure reveals the GSK-1 configuration of the MT-bound inhibited motor.

This raises the question as to when in the MT-dependent HsK5 ATPase cycle GSK-1 binds the motor? The near-occlusion of the proposed GSK-1 binding site by the HsK5-tubulin interaction (Figure 3B) is consistent with the failure of GSK-1-trapped HsK5 to release tightly bound MTs despite multiple washes. This also implies that GSK-1 is unlikely to be able to bind when HsK5 is already MT bound. Given the ATP-like state of the tubulin-binding subdomain, we speculate that GSK-1 could bind to the ADP.Pi state of HsK5 (Figure 3F), which was previously shown to be similar to the ATP-like state (Goulet et al., 2014), but which likely mediates MT release (Milic et al., 2014). GSK-1 binding promotes rebinding of the motor to the MT and forces nucleotide expulsion.

The inhibition mechanism of GSK-1 contrasts with the many inhibitors of HsK5 that bind loop5 but which inhibit the motor by preventing tight MT binding (Crevel et al., 2004; Kwok et al., 2006). The resulting loose MT binding of HsK5 likely makes it easier for another mitotic motor—kinesin-12—to substitute for HsK5 during mitosis in HsK5-knockdown cells (Sturgill et al., 2016), and may offer one explanation for the low efficacy of HsK5-targeting drugs in clinical trials. GSK-1 traps HsK5 close to the centrosome in mitotic cells (Tarui et al., 2014), suggesting that inhibitors, such as GSK-1 could actively block other motors from taking over and thereby promote defects in mitosis, CIN induction, and/or cell death via different routes (Funk et al., 2016) than have previously been characterized for loop5 HsK5 inhibitors. Our work sheds important light on the mechanism of HsK5 inhibition by GSK-1 that will contribute to the development of novel Eg5 inhibition mechanisms in the cellular context. It further reinforces the importance of exploring MT-trapping inhibitors of other mitotic kinesins for the effective disruption of mitosis (Dumas et al., 2019; Locke et al., 2017; Wu et al., 2013).

STAR★METHODS

Detailed methods are provided in the online version of this paper and include the following:

- KEY RESOURCES TABLE
- LEAD CONTACT AND MATERIAL AVAILABILITY
- EXPERIMENTAL MODEL AND SUBJECT DETAILS
- METHOD DETAILS
  - Protein Expression and Purification
  - ATPase Assay
  - MT Gliding Assay
  - MT Depolymerisation Assay
  - Cryo-EM Sample Preparation and Data Collection
  - Cryo-EM Data Processing
  - Atomic Model Calculation
  - Computation of cryo-EM Difference Maps
  - Identification of GSK-1 Binding Sites
  - Modelling GSK-1 in the Binding Site
- QUANTIFICATION AND STATISTICAL ANALYSIS
- DATA AND CODE AVAILABILITY

SUPPLEMENTAL INFORMATION

Supplemental Information can be found online at https://doi.org/10.1016/j.str.2020.01.013.

ACKNOWLEDGMENTS

A.P and C.A.M. were supported by a grant from Worldwide Cancer Research, U.K. (16-0037), A.D.C. and A.S. were supported by PhD studentships from the Medical Research Council, U.K. (MR/J003867/1 and MR/N013867/1, respectively) and funds to A.D.C. from the Wellcome Trust (101311-10), M.T. is supported by a grant from the Wellcome Trust, U.K. (209250/Z/17/Z and 208398/Z/17/Z). EM data were collected on equipment funded by the Wellcome Trust, U.K. (202679/Z/16/Z and 206166/Z/17/Z). The authors thank the Birkbeck EM community and Dr Agnel Joseph for helpful discussions.

AUTHOR CONTRIBUTIONS

A.P. and A.S. conducted the experiments and analyzed the results. A.D.C. provided computational methods and guidance. M.T. and C.A.M. supervised the

work. All authors were involved in designing the experiments and writing the paper.

DECLARATION OF INTERESTS

The authors declare no competing interests.

Received: October 4, 2019
Revised: December 12, 2019
Accepted: January 28, 2020
Published: February 20, 2020

REFERENCES

Cao, L., Wang, W., Jiang, Q., Wang, C., Knossow, M., and Gigant, B. (2014). The structure of apo-kinesin bound to tubulin links the nucleotide cycle to movement. Nat. Commun. *5*, 5364.

Chandrasekaran, G., Tatrai, P., and Gergely, F. (2015). Hitting the brakes: targeting microtubule motors in cancer. Br. J. Cancer *113*, 693–698.

Chattopadhyay, S., Stewart, A.L., Mukherjee, S., Huang, C., Hartwell, K.A., Miller, P.G., Subramanian, R., Carmody, L.C., Yusuf, R.Z., Sykes, D.B., et al. (2015). Niche-based screening in multiple myeloma identifies a kinesin-5 inhibitor with improved selectivity over hematopoietic progenitors. Cell Rep. *10*, 755–770.

Chen, G.Y., Cleary, J.M., Asenjo, A.B., Chen, Y., Mascaro, J.A., Arginteanu, D.F.J., Sosa, H., and Hancock, W.O. (2019). Kinesin-5 promotes microtubule nucleation and assembly by stabilizing a lattice-competent conformation of tubulin. Curr. Biol. *29*, 2259–2269.e4.

Chen, G.Y., Kang, Y.J., Gayek, A.S., Youyen, W., Tuzel, E., Ohi, R., and Hancock, W.O. (2017). Eg5 inhibitors have contrasting effects on microtubule stability and metaphase spindle integrity. ACS Chem. Biol. *12*, 1038–1046.

Chen, Y., and Hancock, W.O. (2015). Kinesin-5 is a microtubule polymerase. Nat. Commun. *6*, 8160.

Cochran, J.C., Sontag, C.A., Maliga, Z., Kapoor, T.M., Correia, J.J., and Gilbert, S.P. (2004). Mechanistic analysis of the mitotic kinesin Eg5. J. Biol. Chem. *279*, 38861–38870.

Cook, A., D., Manka, S., W., Wang, S., Moores, C., A., and Atherton, J. (2020). A microtubule RELION-based pipeline for cryo-EM Image processing. J. Struct. Biol. *209*, https://doi.org/10.1016/j.jsb.2019.10.004.

Crevel, I.M., Alonso, M.C., and Cross, R.A. (2004). Monastrol stabilises an attached low-friction mode of Eg5. Curr. Biol. *14*, R411–R412.

Dumas, M.E., Chen, G.Y., Kendrick, N.D., Xu, G., Larsen, S.D., Jana, S., Waterson, A.G., Bauer, J.A., Hancock, W., Sulikowski, G.A., et al. (2019). Dual inhibition of Kif15 by oxindole and quinazolinedione chemical probes. Bioorg. Med. Chem. Lett. *29*, 148–154.

Farabella, I., Vasishtan, D., Joseph, A.P., Pandurangan, A.P., Sahota, H., and Topf, M. (2015). TEMPy: a Python library for assessment of three-dimensional electron microscopy density fits. J. Appl. Crystallogr. *48*, 1314–1323.

Funk, L.C., Zasadil, L.M., and Weaver, B.A. (2016). Living in CIN: mitotic infidelity and its consequences for tumor promotion and suppression. Dev. Cell *39*, 638–652.

Good, J.A., Skoufias, D.A., and Kozielski, F. (2011). Elucidating the functionality of kinesins: an overview of small molecule inhibitors. Semin. Cell Dev. Biol. *22*, 935–945.

Goulet, A., Major, J., Jun, Y., Gross, S.P., Rosenfeld, S.S., and Moores, C.A. (2014). Comprehensive structural model of the mechanochemical cycle of a mitotic motor highlights molecular adaptations in the kinesin family. Proc. Natl. Acad. Sci. U S A *111*, 1837–1842.

Goulet, A., and Moores, C. (2013). New insights into the mechanism of force generation by kinesin-5 molecular motors. Int. Rev. Cell Mol. Biol. *304*, 419–466.

Groen, A.C., Needleman, D., Brangwynne, C., Gradinaru, C., Fowler, B., Mazitschek, R., and Mitchison, T.J. (2008). A novel small-molecule inhibitor reveals a possible role of kinesin-5 in anastral spindle-pole assembly. J. Cell Sci. *121*, 2293–2300.

Houston, D.R., and Walkinshaw, M.D. (2013). Consensus docking: improving the reliability of docking in a virtual screening context. J. Chem. Inf. Model. *53*, 384–390.

Huang, B. (2009). MetaPocket: a meta approach to improve protein ligand binding site prediction. OMICS *13*, 325–330.

Jones, G., Willett, P., Glen, R.C., Leach, A.R., and Taylor, R. (1997). Development and validation of a genetic algorithm for flexible docking. J. Mol. Biol. *267*, 727–748.

Joseph, A.P., Malhotra, S., Burnley, T., Wood, C., Clare, D.K., Winn, M., and Topf, M. (2016). Refinement of atomic models in high resolution EM reconstructions using Flex-EM and local assessment. Methods *100*, 42–49.

Komlodi-Pasztor, E., Sackett, D.L., and Fojo, T. (2013). Tales of how great drugs were brought down by a flawed rationale—response. Clin. Cancer Res. *19*, 1304.

Kreuzer, K.N., and Jongeneel, C.V. (1983). *Escherichia coli* phage T4 topoisomerase. Methods Enzymol. *100*, 144–160.

Kwok, B.H., Kapitein, L.C., Kim, J.H., Peterman, E.J., Schmidt, C.F., and Kapoor, T.M. (2006). Allosteric inhibition of kinesin-5 modulates its processive directional motility. Nat. Chem. Biol. *2*, 480–485.

Locke, J., Joseph, A.P., Pena, A., Mockel, M.M., Mayer, T.U., Topf, M., and Moores, C.A. (2017). Structural basis of human kinesin-8 function and inhibition. Proc. Natl. Acad. Sci. U S A *114*, E9539–E9548.

Luo, L., Carson, J.D., Molnar, K.S., Tuske, S.J., Coales, S.J., Hamuro, Y., Sung, C.M., Sudakin, V., Auger, K.R., Dhanak, D., et al. (2008). Conformation-dependent ligand regulation of ATP hydrolysis by human KSP: activation of basal hydrolysis and inhibition of microtubule-stimulated hydrolysis by a single, small molecule modulator. J. Am. Chem. Soc. *130*, 7584–7591.

Luo, L., Parrish, C.A., Nevins, N., McNulty, D.E., Chaudhari, A.M., Carson, J.D., Sudakin, V., Shaw, A.N., Lehr, R., Zhao, H., et al. (2007). ATP-competitive inhibitors of the mitotic kinesin KSP that function via an allosteric mechanism. Nat. Chem. Biol. *3*, 722–726.

Maier, J.A., Martinez, C., Kasavajhala, K., Wickstrom, L., Hauser, K.E., and Simmerling, C. (2015). ff14SB: improving the accuracy of protein side chain and backbone parameters from ff99SB. J. Chem. Theor. Comput. *11*, 3696–3713.

Manka, S.W., and Moores, C.A. (2018). The role of tubulin-tubulin lattice contacts in the mechanism of microtubule dynamic instability. Nat. Struct. Mol. Biol. *25*, 607–615.

Mann, B.J., and Wadsworth, P. (2019). Kinesin-5 regulation and function in mitosis. Trends Cell Biol. *29*, 66–79.

Mayer, T.U., Kapoor, T.M., Haggarty, S.J., King, R.W., Schreiber, S.L., and Mitchison, T.J. (1999). Small molecule inhibitor of mitotic spindle bipolarity identified in a phenotype-based screen. Science *286*, 971–974.

Milic, B., Andreasson, J.O., Hancock, W.O., and Block, S.M. (2014). Kinesin processivity is gated by phosphate release. Proc. Natl. Acad. Sci. U S A *111*, 14136–14140.

Mitchison, T.J. (2012). The proliferation rate paradox in antimitotic chemotherapy. Mol. Biol. Cell *23*, 1–6.

Parke, C.L., Wojcik, E.J., Kim, S., and Worthylake, D.K. (2010). ATP hydrolysis in Eg5 kinesin involves a catalytic two-water mechanism. J. Biol. Chem. *285*, 5859–5867.

Pettersen, E.F., Goddard, T.D., Huang, C.C., Couch, G.S., Greenblatt, D.M., Meng, E.C., and Ferrin, T.E. (2004). UCSF Chimera—a visualization system for exploratory research and analysis. J. Comput. Chem. *25*, 1605–1612.

Pintilie, G.D., Zhang, J., Goddard, T.D., Chiu, W., and Gossard, D.C. (2010). Quantitative analysis of cryo-EM density map segmentation by watershed and scale-space filtering, and fitting of structures by alignment to regions. J. Struct. Biol. *170*, 427–438.

Rath, O., and Kozielski, F. (2012). Kinesins and cancer. Nat. Rev. Cancer *12*, 527–539.

Rohou, A., and Grigorieff, N. (2015). CTFFIND4: fast and accurate defocus estimation from electron micrographs. J. Struct. Biol. *192*, 216–221.

Salentin, S., Schreiber, S., Haupt, V.J., Adasme, M.F., and Schroeder, M. (2015). PLIP: fully automated protein-ligand interaction profiler. Nucleic Acids Res. *43*, W443–W447.

Sali, A., and Blundell, T.L. (1993). Comparative protein modelling by satisfaction of spatial restraints. J. Mol. Biol. *234*, 779–815.

Schindelin, J., Arganda-Carreras, I., Frise, E., Kaynig, V., Longair, M., Pietzsch, T., Preibisch, S., Rueden, C., Saalfeld, S., Schmid, B., et al. (2012). Fiji: an open-source platform for biological-image analysis. Nat. Methods *9*, 676–682.

Shang, Z., Zhou, K., Xu, C., Csencsits, R., Cochran, J.C., and Sindelar, C.V. (2014). High-resolution structures of kinesin on microtubules provide a basis for nucleotide-gated force-generation. Elife *3*, e04686.

Shen, M.Y., and Sali, A. (2006). Statistical potential for assessment and prediction of protein structures. Protein Sci. *15*, 2507–2524.

Sturgill, E.G., Norris, S.R., Guo, Y., and Ohi, R. (2016). Kinesin-5 inhibitor resistance is driven by kinesin-12. J. Cell Biol. *213*, 213–227.

Tarui, Y., Chinen, T., Nagumo, Y., Motoyama, T., Hayashi, T., Hirota, H., Muroi, M., Ishii, Y., Kondo, H., Osada, H., et al. (2014). Terpendole E and its derivative inhibit STLC- and GSK-1-resistant Eg5. Chembiochem *15*, 934–938.

Topf, M., Lasker, K., Webb, B., Wolfson, H., Chiu, W., and Sali, A. (2008). Protein structure fitting and refinement guided by cryo-EM density. Structure *16*, 295–307.

Toropova, K., Mladenov, M., and Roberts, A.J. (2017). Intraflagellar transport dynein is autoinhibited by trapping of its mechanical and track-binding elements. Nat. Struct. Mol. Biol. *24*, 461–468.

Trott, O., and Olson, A.J. (2010). AutoDock Vina: improving the speed and accuracy of docking with a new scoring function, efficient optimization, and multithreading. J. Comput. Chem. *31*, 455–461.

Uchimura, S., Fujii, T., Takazaki, H., Ayukawa, R., Nishikawa, Y., Minoura, I., Hachikubo, Y., Kurisu, G., Sutoh, K., Kon, T., et al. (2015). A flipped ion pair at the dynein-microtubule interface is critical for dynein motility and ATPase activation. J. Cell Biol. *208*, 211–222.

Ulaganathan, V., Talapatra, S.K., Rath, O., Pannifer, A., Hackney, D.D., and Kozielski, F. (2013). Structural insights into a unique inhibitor binding pocket in kinesin spindle protein. J. Am. Chem. Soc. *135*, 2263–2272.

Wu, J., Mikule, K., Wang, W., Su, N., Petteruti, P., Gharahdaghi, F., Code, E., Zhu, X., Jacques, K., Lai, Z., et al. (2013). Discovery and mechanistic study of a small molecule inhibitor for motor protein KIFC1. ACS Chem. Biol. *8*, 2201–2208.

Yan, Y., Sardana, V., Xu, B., Homnick, C., Halczenko, W., Buser, C.A., Schaber, M., Hartman, G.D., Huber, H.E., and Kuo, L.C. (2004). Inhibition of a mitotic motor protein: where, how, and conformational consequences. J. Mol. Biol. *335*, 547–554.

Yokoyama, H., Sawada, J., Katoh, S., Matsuno, K., Ogo, N., Ishikawa, Y., Hashimoto, H., Fujii, S., and Asai, A. (2015). Structural basis of new allosteric inhibition in kinesin spindle protein Eg5. ACS Chem. Biol. *10*, 1128–1136.

Zheng, S.Q., Palovcak, E., Armache, J.P., Verba, K.A., Cheng, Y., and Agard, D.A. (2017). MotionCor2: anisotropic correction of beam-induced motion for improved cryo-electron microscopy. Nat. Methods *14*, 331–332.

Zivanov, J., Nakane, T., Forsberg, B.O., Kimanius, D., Hagen, W.J., Lindahl, E., and Scheres, S.H. (2018). New tools for automated high-resolution cryo-EM structure determination in RELION-3. eLife *7*, https://doi.org/10.7554/eLife.42166.

STAR★METHODS

KEY RESOURCES TABLE

REAGENT or RESOURCE	SOURCE	IDENTIFIER
Bacterial and Virus Strains		
Competent BL21-CodonPlus (DE3)-RIL *E. coli*	Agilent Technologies	Cat# 230245
Biological Samples		
Porcine brain tubulin	Cytoskeleton Inc.	Cat# T240
X-rhodamine labeled, bovine brain tubulin	Cytoskeleton Inc.	Cat# TL620M
Biotin-labeled, porcine brain tubulin	Cytoskeleton Inc.	Cat# T333P
Chemicals, Peptides, and Recombinant Proteins		
GSK-1	Santa Cruz Biotechnology	Cat# SC-221578
STLC	Sigma	Cat# 164739
Human kinesin-5 motor domain (1-368)	This study	N/A
Human kinesin-5 motor domain + SNAPf	This study	N/A
Human kinesin-5 motor domain-E116A	This study	N/A
Human kinesin-5 motor domain-E118A	This study	N/A
Human kinesin-5 motor domain-R119A	This study	N/A
Human kinesin-5 motor domain-W127A	This study	N/A
Human kinesin-5 motor domain-Y211A	This study	N/A
Human kinesin-5 motor domain-I299F	This study	N/A
Human kinesin-5 motor domain-A356T	This study	N/A
Human kinesin-5 motor domain-I299A+A356T	This study	N/A
Human kinesin-5 motor domain-I299F + SNAPf	This study	N/A
Human kinesin-5 motor domain-A356T + SNAPf	This study	N/A
Deposited Data		
HsK5+MT+GSK-1 EM density	This study	EMDB 10421
HsK5+MT+GSK-1 model, conformation 1	This study	PDB 6TA3
HsK5+MT+GSK-1 model, conformation 2	This study	PDB 6TIW
HsK5+MT+AMPPNP EM density	This study	EMDB 10422
HsK5+MT+AMPPNP model	This study	PDB 6TA4
HsK5-PVZB1194	Yokoyama et al., 2015	PDB 3WPN
HsK5-AMPPNP	Parke et al., 2010	PDB 3HQD
α- and β-tubulin GMPCPP MT	Manka and Moores, 2018	PDB: 6EVW
Oligonucleotides		
Primers for HsK5 in pNIC28-Bsa4: TACTTCCAATC CATGGCTTCTCAGCCCAACTC, TATCCACCTTT ACTGTTATTATTTTTGGTTGACTT	Eurofins Genomics	N/A
Primers for HsK5 SNAPf in pNIC28-Bsa4 CATTTCGCAGTCTTTGTCCATTTTTTGGTTGAC, CCCGAAGTCAACCAAAAAATGGACAAAGAC TGCGAAATG,GCCTGGGCTGGGTTAATAACAGTA AAGGTGGATACGGGGCTCCTTCCTCCGCCATA TGGCTGCCGCGCGGCACCAGG,	Eurofins Genomics	N/A
Human kinesin-5 motor domain-E116A TTCACAATGGCAGGTGAACGTTCTCCCAAT, ACGTTCACCTGCCATTGTGAATGTCTTACC	Eurofins Genomics	N/A
Human kinesin-5 motor domain-E118A ATGGAAGGTGCACGTTCTCCCAATGAGGAA, GGGAGAACGTGCACCTTCCATTGTGAATGT	Eurofins Genomics	N/A

(*Continued on next page*)

Continued

REAGENT or RESOURCE	SOURCE	IDENTIFIER
Human kinesin-5 motor domain-R119A GAAGGTGAAGCTTCTCCCAATGAGGAATAT, ATTGGGAGAAGCTTCACCTTCCATTGTGAA	Eurofins Genomics	N/A
Human kinesin-5 motor domain-W127A GAATATACGGCGGAAGAAGATCCGCTGGCA, ATCTTCTTCCGCCGTATATTCCTCATTGGG	Eurofins Genomics	N/A
Human kinesin-5 motor domain-Y211A GATGAAGTACCCCAAATATTGGAAAAGGGA, CAATATTTGGGGTACTTCATCCTTGTTATG	Eurofins Genomics	N/A
Human kinesin-5 motor domain-I299F GGTCGCGTATTTACCGCCTTAGTGGAAAAG, TAAGGCGGTAAATACGCGACCCAGGGTGAG	Eurofins Genomics	N/A
Human kinesin-5 motor domain-A356T GCACACCGCACAAAGAACATACTGAACAAA, TATGTTCTTTGTGCGGTGTGCATATTCCAG	Eurofins Genomics	N/A
Recombinant DNA		
pNIC28-Bsa4	Structural Genomics Consortium	https://www.thesgc.org/reagents/vectors
Software and Algorithms		
GraphPad Prism 6.0	GraphPad Software, La Jolla California USA	https://www.graphpad.com/scientific-software/prism/
Fiji	(Schindelin et al., 2012)	https://fiji.sc
MotionCor2	(Zheng et al., 2017)	https://emcore.ucsf.edu/ucsf-motioncor2
RELION-3.0	(Zivanov et al., 2018)	https://www3.mrc-lmb.cam.ac.uk/relion//index.php?title=Main_Page
MiRP	(Cook et al., 2020)	https://github.com/moores-lab/MiRP
MODELLER v9.21	(Sali and Blundell, 1993)	https://salilab.org/modeller/
Chimera	(Pettersen et al., 2004	https://www.cgl.ucsf.edu/chimera/
Flex-EM	(Topf et al., 2008)	http://topf-group.ismb.lon.ac.uk/flex-em/
TEMPy	(Farabella et al., 2015)	http://tempy.ismb.lon.ac.uk
Metapocket-V2	(Huang, 2009)	https://projects.biotec.tu-dresden.de/metapocket/
AutoDock Vina	(Trott and Olson, 2010)	http://vina.scripps.edu/
GOLD	(Jones et al., 1997)	https://www.ccdc.cam.ac.uk/solutions/csd-discovery/components/gold/

LEAD CONTACT AND MATERIAL AVAILABILITY

Further information and requests for resources and reagents should be directed to and will be fulfilled by the Lead Contact, Carolyn Moores (c.moores@mail.cryst.bbk.ac.uk). All unique/stable reagents generated in this study are available from the Lead Contact without restriction.

EXPERIMENTAL MODEL AND SUBJECT DETAILS

All human kinesin-5 constructs were expressed in *E. coli* BL21-CodonPlus (DE3)-RIL cells.

METHOD DETAILS

Protein Expression and Purification

The human kinesin-5 motor domain (residues Met1–Lys368) was PCR amplified from a codon-optimised synthetic DNA fragment (GeneOracle) and cloned into the pNIC28-Bsa4 vector (from the Structural Genomics Consortium) containing a TEV-cleavable N-terminal His_6-tag. HsK5-SNAPf constructs were also generated in which a C-terminal SNAPf tag was introduced using Gibson assembly. Point mutations were introduced into HsK5 by PCR. Constructs were transformed into *E. coli* BL21-CodonPlus (DE3)-RIL (Agilent Technologies), which were grown in LB media at 37°C to an OD600 of 0.6, cooled to 20°C, induced with 0.5 mM isopropyl β-thiogalactopyranoside (IPTG) and left growing overnight. Cells were harvested by centrifugation and stored at -80°C. Lysis was

carried out in 50 mM Tris-HCl pH 7.6, 500 mM NaCl, 50 mM Imidazole, 0.1 % Triton-X100, 5 mM $MgCl_2$, and complete protease inhibitor mix (Roche). Soluble His_6-tagged HsK5 was bound to a 5ml NTA-nickel column (GE Healthcare) and eluted with an increasing Imidazole gradient (50 mM Tris-HCl pH 7.6, 150 mM NaCl, 50-500 mM Imidazole, 5 mM $MgCl_2$). Protein-containing fractions were pooled, concentrated and loaded on a Superdex 200 Increase gel filtration column (GE Healthcare) in a buffer (20 mM Tris-HCl pH 7.6, 150 mM NaCl, 5mM $MgCl_2$). HsK5-containing fractions were pooled and concentrated using an Amicon Ultra-4 centrifugal filter 30kDa (Millipore), snap-frozen and stored at -80°C. His_6-HsK5-SNAPf constructs were purified in the same way.

ATPase Assay

Paclitaxel-stabilised MTs were polymerised using 50 μM porcine tubulin (Cytoskeleton, Inc.) mixed with polymerization buffer (100 mM MES-KOH pH 6.5, 1 mM $MgCl_2$, 1 mM EGTA, 1 mM DTT, 5 mM GTP) at 37°C for 1 hour. 1 mM Paclitaxel (Calbiochem) in DMSO was added and the sample was incubated for a further hour at 37°C. MTs were kept at room temperature and used after 24 hours.

The steady-state ATPase activities of HsK5 constructs were determined using an enzyme-coupled assay system (Kreuzer and Jongeneel, 1983). The reaction was performed in buffer containing 20 mM Tris-HCl pH 7.6, 150 mM NaCl, 5mM $MgCl_2$, 250 μM NADH, 5 mM phosphoenolpyruvate, 10 U/ml pyruvate kinase and 14 U/ml lactate dehydrogenase and 5mM ATP. To this reaction buffer, varying amounts of paclitaxel-stabilized MTs (up to 2 μM) were added and subsequently mixed with HsK5 at a final concentration of 1.5 μM. Reactions were performed in a 96-well plate with a volume of 100 μl per well. A340 was measured every 10 secs in a SpectraMax Plus 384 Microplate Reader (Molecular Devices) for 10 min at 37°C. ATPase rates were plotted and used to calculate Km for ATP and $K_{1/2}$ for MTs by performing Michaelis-Menten fits in GraphPad Prism 6.0 (GraphPad Software, La Jolla California USA). For GSK-1 (Santa Cruz Biotechnology) inhibition curves using relative activities were determined by setting the rate in the reaction not containing GSK-1 to 100%. In this assay, the same HsK5 concentration were used and 100nM MTs (saturated). The fit to determine the GSK-1 IC_{50} for both the ATPase and the gliding assay data (below) was y = a/(1+((x/b)^c)), where a= Vmax, b = IC_{50}, c = Hill coefficient in GraphPad Prism.

MT Gliding Assay

10 μM HsK5-SNAPf constructs were biotinylated for surface immobilisation by incubating them with 20 μM SNAP-biotin (NEB) in a 50 μl reaction volume at 4°C for 2 hours. Proteins were purified from excess SNAP-biotin by size-exclusion chromatography on a Superdex75 Increase 3.2/300 column using an ÅKTA micro system (GE Healthcare), pre-equilibrated with gel filtration buffer (20 mM Tris-HCl pH 7.6, 150 mM NaCl, 5 mM $MgCl_2$). Fractions (100 μl) were analysed by SDS-PAGE and peak fractions were flash frozen in liquid nitrogen in single-use aliquots and stored at -80°C. Fluorescently-labelled MTs, containing 10% Rhodamine-X tubulin (Cytoskeleton, Inc.), were polymerised in the presence of GTP and stabilised by addition of paclitaxel (as above).

Flow chambers for Total Internal Reflection Fluorescence (TIRF) microscopy were prepared between glass slides, biotin-PEG coverslips (MicroSurfaces Inc.), using double-sided tape. Chambers were sequentially incubated with 1) blocking solution (0.75% Pluronic F-127, 5 mg/ml casein) for 5 min, followed by two washes with B20-TK (20 mM Tris-HCl pH 7.6, 150 mM NaCl, 5 mM $MgCl_2$); 2) 0.5 mg/ml neutravidin for 2 min, followed by two washes with B20-TK; 3) biotinylated motor protein (15 nM) for 2 min, followed by two washes with B20-TK supplemented with 1 mg/ml casein; 4) 0.1 μM Rhodamine-X/Alexa-488 MTs in assay solution (B20-TK supplemented with 1 mg/ml casein, 1 mM Mg-ATP and an oxygen scavenging mix (71 mM β- mercaptoethanol, 20 mM glucose, 300 μM /ml glucose oxidase, 60 μg/ml catalase)). Gliding assays were performed at room temperature by TIRF microscopy as described (Toropova et al., 2017), using an Eclipse Ti-E inverted microscope with a CFI Apo TIRF 1.49 N.A. oil objective, Perfect Focus System, H-TIRF module, LU-N4 laser unit (Nikon) and a quad band filter set (Chroma). Images were recorded with 100 ms exposures on an iXon DU888 Ultra EMCCD camera 3 (Andor), controlled with NIS-Elements AR Software (Nikon). Gliding velocities were determined from kymographs generated using Fiji (Schindelin et al., 2012). The movement of all the sparsely distributed MTs in the fields of view were analysed. For each condition, data from 6 or more movies were analysed.

MT Depolymerisation Assay

MTs containing 10% Rhodamine-X and 10% biotin-labelled tubulin were polymerised with GTP and paclitaxel-stabilised as described above. Chambers for TIRF microscopy (prepared as described above) were sequentially incubated with 1) 0.5 mg/ml neutravidin for 2 min, followed by two washes with assay buffer (20 mM Tris-HCl 7.6, 150 mM NaCl, 5 mM $MgCl_2$, 0.5 mg/ml casein, 20 μM paclitaxel); 2) a 1:100 dilution of MT suspension for 2 min, followed by two washes with assay buffer; 3) Unlabelled 625 nM HsK5 in assay buffer supplemented with 1 mM nucleotide, 50 nM GSK-1, or 50 nM STLC, together with the oxygen scavenging mix (as above). In the control experiment without motor protein, the buffer compositions were as mentioned above but without 20 μM paclitaxel addition. Depolymerisation assays were visualised over 30 min using TIRF microscopy as described above. Depolymerisation rates were determined from kymographs generated using ImageJ. Where necessary, image drift was corrected using StackReg rigid body transformation. Depolymerisation of all the MTs in the fields of view were investigated, including those that were not obviously depolymerizing. For each condition, data from three or more movies were analysed. Statistical analysis between different conditions were carried out using one-way ANOVA and post-hoc Tukey test in GraphPad Prism.

Cryo-EM Sample Preparation and Data Collection

GMPCPP-stabilised MTs were polymerised using 50 μM porcine tubulin (Cytoskeleton, Inc.) mixed with MT polymerization buffer (100 mM MES-KOH pH 6.5, 1 mM $MgCl_2$, 1 mM EGTA, 1 mM DTT, 5 mM GMPCPP (Jena) at 37°C for 30 minutes. The MTs were centrifuged at room temperature 15000g for 5 min, the supernatant was discarded, and the MT pellet was resuspended in the same buffer with vortexing. This was incubated on ice for 3 minutes followed by a further incubation of 30 min at 37°C. MTs were kept at room temperature and used after 24 hours. To form the complex with GSK-1, 25 μM HsK5 was incubated with 10 μM GSK-1 for 15 minutes on ice, followed by incubation with 10 μM MTs for another 15 minutes at room temperature before vitrification. To form the complex with AMPPNP, 25 μM HsK5 was incubated with 20 mM AMPPNP and kept on ice for 15 min, followed by incubation with 10 μM MTs before vitrification.

Carbon grids (C-Flat 2/2 grids; Protochips) were glow-discharged in air, and 4 μL of each sample was applied. The grids were blotted with a final blotting time of 2.5 s and vitrified in liquid ethane using a Vitrobot Mark III (FEI) at 25°C and 100% humidity. Low dose movies of the AMPPNP complex were collected manually on a 300-kV Tecnai G2 Polara microscope (FEI) equipped with a Quantum energy filter and K2 Summit direct electron detector (Gatan) in counting mode at a pixel size of 1.39 Å. The total exposure was ~30e-/Å2 over 10 seconds at 20 frames/sec with a defocus range between ~0.5 and 3.5 μm for AMPPNP. Data for the GSK-1 complex were collected on a ThermoFisher Titan Krios using EPU operated at 300-kV equipped with a K2 Summit direct electron detector (Gatan) in counting mode at a pixel size of 1.09 Å. The total exposure was ~45e-/Å2 over 8 seconds at 32 frames/sec, with a defocus range between ~-1 and -3.5 μm.

Cryo-EM Data Processing

Movie frames were aligned using MotionCor2 (Zheng et al., 2017) to generate full dose and dose-weighted micrographs. The CTF of full dose micrographs was calculated with CTFFIND-4.1 (Rohou and Grigorieff, 2015) in RELION-3.0 (Zivanov et al., 2018). The start-end coordinates of MTs were manually picked in RELION, and MT particles extracted every 82 Å from dose-weighted micrographs. GSK-1 dataset size: 2,261 movies yielding 507, 219 MT segments; AMPPNP dataset size: 119 movies yielding 43, 714 MT segments. Classification and alignment of MT particles, followed by 3D reconstruction of 14-3 MTs was performed using RELION and custom scripts, according to the MiRP procedure (Cook et al., 2019). Briefly, supervised 3D classification was used to select 14-3 MTs, then MT Rot angle assignment, X/Y shift smoothing, and seam checking steps from MiRP were used to align asymmetric 14-3 MT particles. 3D auto-refinement was then performed, followed by per-particle CTF refinement and Bayesian polishing (Zivanov et al., 2018). A final 3D auto-refinement was performed with helical symmetry applied.

Atomic Model Calculation

For the HsK5-GSK-1 cryo-EM structure, the crystal structure of the human kinesin-5 motor domain in complex with PVZB1194 in a MT-free state (PDB: 3WPN) was used as a starting point for initial model building. However, this structure is missing information about helix-α3, helix-α4, switch I, switch II, loop5, P-loop and loop2 due to flexibility. Having a starting conformation closer to the target structure can improve the speed and accuracy of density-based model refinement (Joseph et al., 2016), and therefore a second template model - human kinesin-5 in an AMPPNP bound state (PDB: 3HQD) was combined with 3WPN to create a hybrid model using MODELLER v9.21 (Sali and Blundell, 1993). This conserved the local conformation in 3WPN, except for the missing regions which were modelled based on 3HQD. The best model was then selected from multiple models using MODELLER DOPE score (Shen and Sali, 2006) and was used as an initial model for refinement in the HsK5-GSK-1 cryo-EM density. 3HQD was used as an initial model for refinement in the HsK5-AMPPNP cryo-EM density. In both reconstructions, a cryo-EM derived MT-GMPCPP structure of α- and β-tubulin was used (PDB: 6EVW).

For both HsK5 reconstructions, density corresponding to HsK5 and αβ-tubulin was segmented using the Segger tool (Pintilie et al., 2010) implemented in Chimera (Pettersen et al., 2004), and segmented densities were used to refine the models. Initial models were rigidly fitted into their respective maps with the Chimera *fit-in-map* function, and real-space refinement was carried out in a hierarchical fashion using Flex-EM (Topf et al., 2008). At each stage of the refinement models were assessed using the TEMPy SMOC score (Farabella et al., 2015).

Computation of cryo-EM Difference Maps

A map of the differences between the HsK5-GSK-1 reconstruction and HsK5-AMPPNP reconstruction was calculated using TEMPy (Farabella et al., 2015). To identify potential GSK-1 binding conformations from molecular docking software output (detailed below), the cross-correlation coefficient (CCC) was calculated between these conformations and both the difference map and the overall HsK5-GSK-1 reconstruction.

Identification of GSK-1 Binding Sites

To identify possible GSK-1 binding sites, a protocol, combining three methodologies, was used (Figure S3A). First, the Metapocket-V2 server (Huang, 2009), which uses eight pocket prediction methods, was used to assign potential solvent accessible binding pockets to the refined HsK5 model. Second, blind ligand docking was conducted with AutoDock Vina (Trott and Olson, 2010) in order to identify pockets that would exhibit a favourable interaction with GSK-1. The latter was performed with a box size that encompassed the entire protein. The *exhaustiveness* option was set to 10, *number of modes* set to 20, and the *maximum energy difference* set to 3 kcal/mol. To adequately explore the large search space, we ran AutoDock Vina 100 times and the results from

these runs were merged. Redundant conformations (<= 2 Å RMSD) were grouped and represented by the conformation that had the best energy score. Unique conformations were clustered by their centroid to identify potential binding sites. Third, the difference map was used to identify density in the reconstruction that was not accounted for by the HsK5 model. The consensus between all three methods (pocket prediction, blind ligand docking and difference map localisation) was used to identify the GSK-1 binding site. A visual inspection of the area within the full map also clearly showed density not accounted for by the model, which corresponded to the approximate size of the expected ligand density.

Modelling GSK-1 in the Binding Site

Consensus Ligand Docking

A two-stage protocol was used to dock GSK-1 to the inhibitor binding site. First, GSK-1 was docked into the identified binding site using focused docking with three scoring functions implemented in GOLD (Jones et al., 1997): Chemscore, Goldscore and ChemPLP. For each run, a binding site radius of 12 Å was used, the *generate diverse solutions* option was on, and the output was set to yield 100 conformations. For individual runs, redundant docking conformations (<= 2 Å RMSD) were grouped and represented by the conformation with the best score. Since it has been shown that consensus predictions can increase the accuracy of docking (Houston and Walkinshaw, 2013), only conformations predicted by all three scoring functions were analysed.

Applying Density Constraints

Consensus conformations (<= 2 Å RMSD) were then clustered and the CCC between each conformation and both the full map and difference map was calculated using TEMPy. The best conformation was selected as having the highest average CCC with both full and difference maps.

Local Refinement in the Density

Since the best-scoring conformations did not adequately fit the density, we hypothesised that this was due to the sidechain positions within the binding site being incorrectly placed in the initial model. Therefore, the rigid fit of the best scoring pose was further optimised into the full density map around the binding site using the Chimera *fit-in-map* function, and the side chain atoms of residues that lined the binding site (within 5 Å of the ligand) were refined in the presence of the ligand using an all-atom refinement with Flex-EM (Topf et al., 2008), while keeping the ligand rigid.

Focused Docking

The second stage of ligand docking aimed to identify a ligand conformation that is well correlated with the density map, given the sidechain position around the pocket. Again, three of the scoring functions implemented in GOLD were used along with AutoDock Vina to dock the ligand into the model from the previous step. This step was done to explore new potential, physically plausible GSK-1 conformations within the defined pocket. For each GOLD run, a tight radius of 6 Å was used, the *generate diverse solutions* option was on and the output was set to yield 100 conformations. For AutoDock Vina, a box-size of 12 $Å^3$ was used, *num_modes* was set to 20, and all other settings were used as default.

Energy Minimisation

Conformations predicted by all four programs were individually assessed based on CCC to the identified unaccounted-for density within the full map using Chimera. Sidechains of residues for which at least one atom was within 5 Å of any GSK-1 atoms (of the conformations with the highest CCC to the full map) were energy minimised with the AMBER forcefield (Maier et al., 2015) using Chimera.

QUANTIFICATION AND STATISTICAL ANALYSIS

Analysis of TIRF images was performed using Fiji (Schindelin et al., 2012) and data were plotted and statistical analysis was performed using GraphPad Prism. Statistical details of experiments and tests applied are included in the STAR Methods DETAIL text and in figure legends. One-way ANOVA and post-hoc Tukey test were used for statistical analysis and data are plotted as mean and S.D. as indicated in figure legends. For each experiment, n values are given in the figure legends and STAR Methods DETAIL section. Local goodness-of-fit for the atomic models within the map during flexible fitting was assessed using the SMOC score (Joseph et al., 2016), implemented in TEMPy (Farabella et al., 2015). The SMOC score gives an assessment of the correlation of a sliding window of 9 residues of the atomic model with the cryo-EM map. Global CCC was calculated with both TEMPy and Chimera (Pettersen et al., 2004).

DATA AND CODE AVAILABILITY

The cryo-EM reconstructions that support the findings of this study have been deposited in the Electron Microscopy Data Bank (accession nos. 10421 (GSK-1) and 10422 (AMPPNP)). The docked coordinates reported in this paper have been deposited in the Protein Data Bank (accession nos. 6TA3 (GSK-1, conformation 1), 6TIW (GSK-1, conformation 2) and 6TA4 (AMPPNP)).

Structure, Volume *28*

Supplemental Information

Structure of Microtubule-Trapped Human Kinesin-5 and Its Mechanism of Inhibition Revealed Using Cryoelectron Microscopy

Alejandro Peña, Aaron Sweeney, Alexander D. Cook, Maya Topf, and Carolyn A. Moores

Structure of microtubule-trapped human kinesin-5 and its mechanism of inhibition revealed using cryo-EM

Alejandro Peña [1,2], Aaron Sweeney [1], Alexander D. Cook [1], Maya Topf [1] and Carolyn A. Moores [1]*

[1] Institute of Structural and Molecular Biology, Birkbeck College, London WC1E 7HX, U.K.
[2] Current address: Department of Life Sciences, Medical Research Council Centre for Molecular Bacteriology & Infection, Imperial College London, London SW7 2BX, UK

* Corresponding Author/Lead Contact
Carolyn A. Moores; ORCID ID: 0000-0001-5686-6290
E-mail: c.moores@mail.cryst.bbk.ac.uk

Supplemental Figures

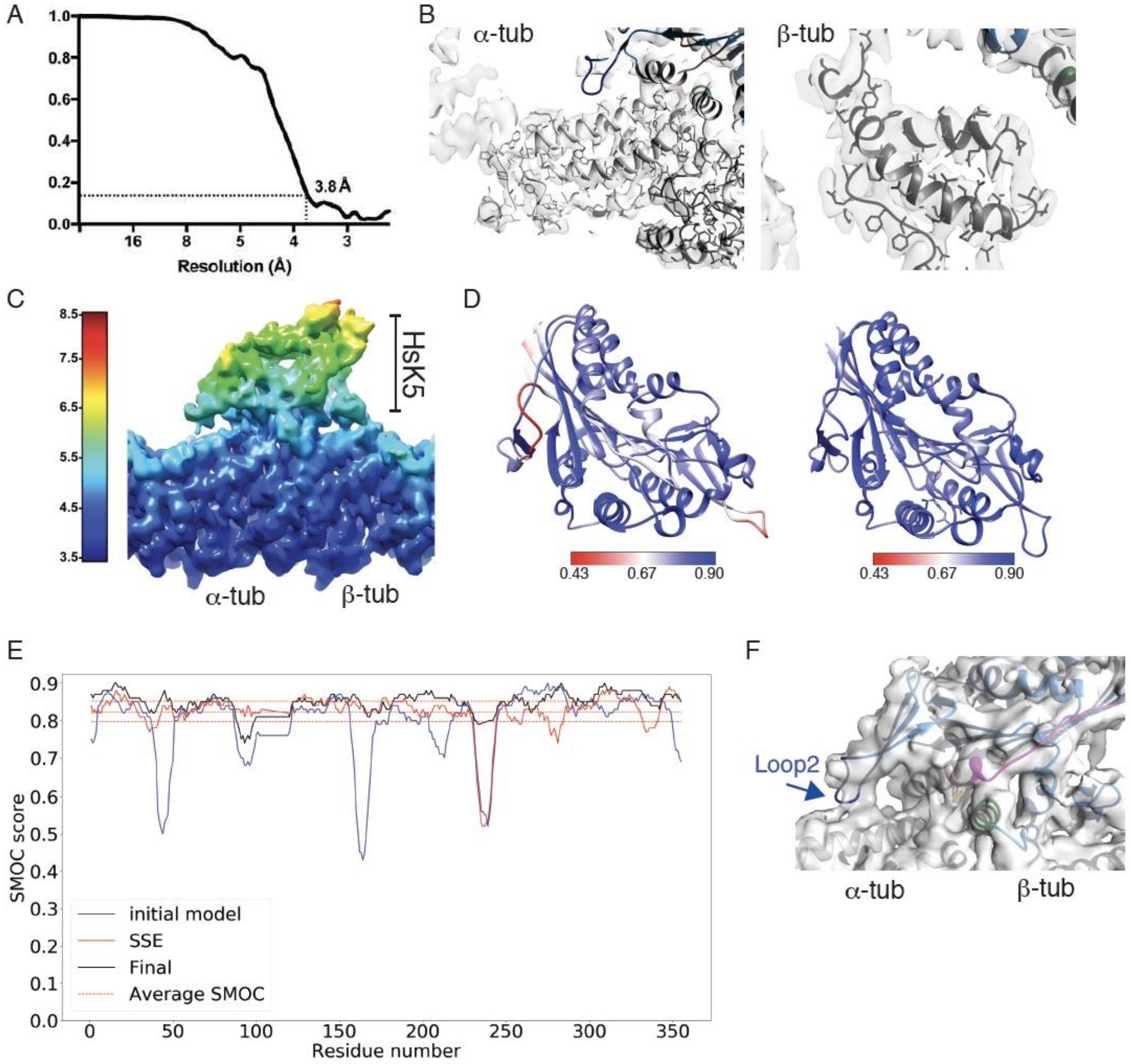

Figure S1 Related to Figure 2. Evaluation of the MT-trapped HsK5 bound by GSK-1 cryo-EM reconstruction. (A) FSC curve for the overall reconstruction estimated to be 3.8 Å by the 0.143 criterion; (B) Visualization of near-atomic resolution features in the MT regions of the density supports the near-atomic resolution estimate of the reconstruction; (C) Depiction of the local resolution estimate in the reconstruction using RELION (Zivanov et al., 2018) indicates the presence of a resolution gradient between the MT and the kinesin; (D) The calculated HsK5 atomic model before (left) and after (right) Flex-EM refinement in the MT-bound HsK5-GSK-1 cryo-EM density were assessed with the TEMPy SMOC score (Joseph et al., 2016) used here to color the final coordinates, illustrating the high quality of the fit; (E) Plot of SMOC scores calculated sequentially for the initial model, after refinement of secondary structural elements (SSEs) and for the final model; (F) At inclusive density thresholds, connectivity is seen between HsK5 loop2 and α-tubulin (arrow), although this more inclusive density was not included as part of the modelling procedure.

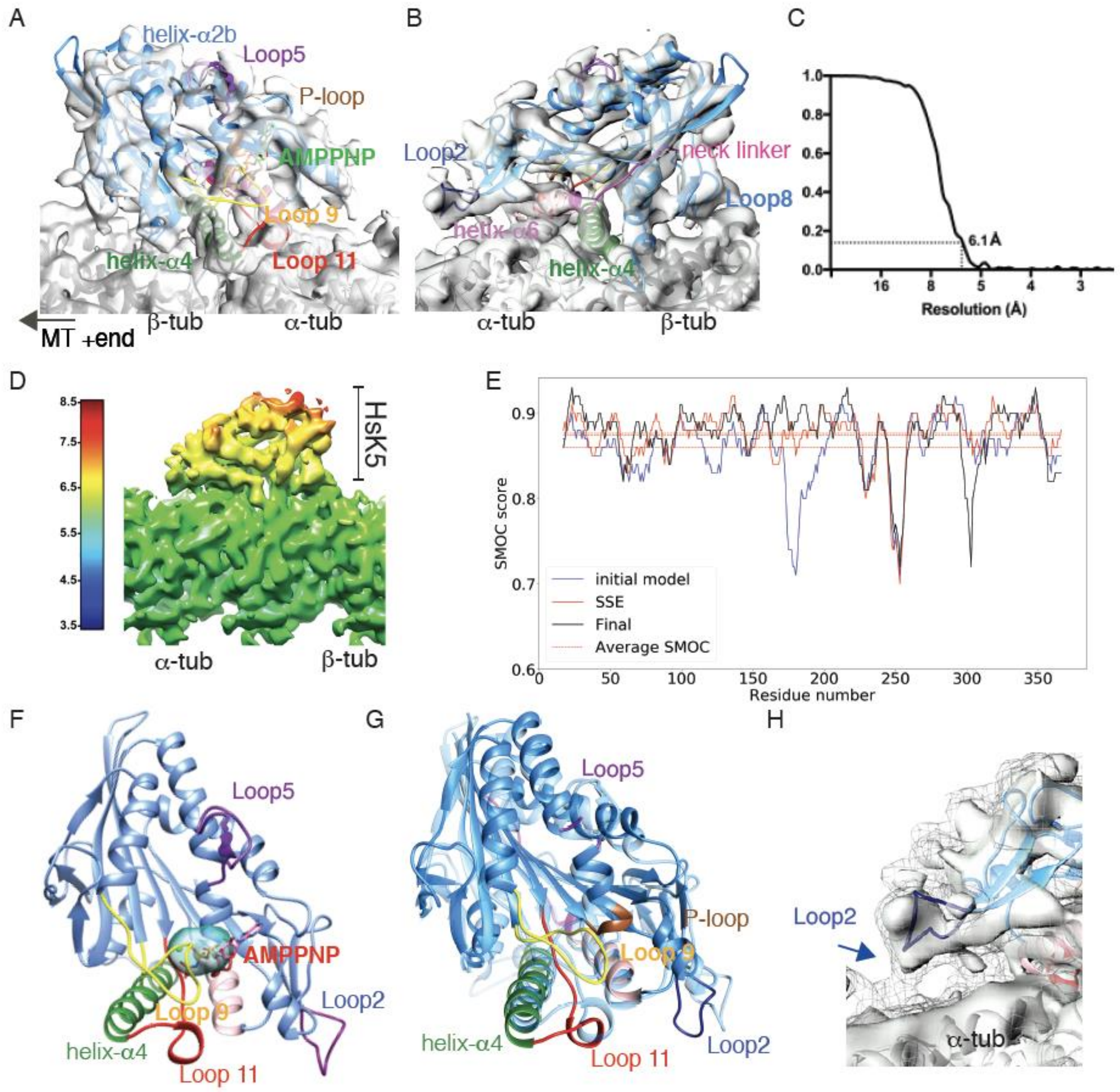

Figure S2 Related to Figure 2. Evaluation of the MT-bound HsK5-AMPPNP cryo-EM reconstruction resolution. (A) An asymmetric unit from the HsK5-AMPPNP-MT reconstruction contoured to show secondary structural elements, and viewed with the MT plus end on the left; the cryo-EM densities are shown as a grey surface representation, α- and β-tubulin are shown in light and dark grey ribbons respectively and the HsK5 model is shown in coloured ribbons with individual secondary structural elements highlighted (helix-α4, green; P-loop, brown; switch 1, loop 9, yellow; switch 2, loop 11, red; loop5, purple; bound nucleotide (modelled as AMPPNP, based on prior knowledge (Goulet et al., 2014) and consistent with sample preparation conditions but not directly identified as such from the density) is shown in stick representation); (B) An asymmetric unit from the HsK5-AMPPNP-MT reconstruction contoured as in (A) viewed with the MT plus end on the right (helix-α6, pink; loop2, dark blue; neck-linker, fuchsia); (C) FSC curve for the overall reconstruction estimated to be 6.1 Å by the 0.143 criterion; (D) Depiction of the local resolution estimate in the reconstruction using RELION (Zivanov et al., 2018); (E) Plot of SMOC scores calculated sequentially for the initial model, after refinement of SSEs and for the final model; note the different scale compared to Figure S1E for ease of visualization; (F) Calculated map vs protein model difference (in turquoise) identifies density corresponding to bound nucleotide

(particularly the phosphate moieties, here modelled as AMPPNP) at the HsK5 nucleotide binding site; the AMPPNP-bound HsK5 conformation is shown in blue ribbons with individual secondary structural elements highlighted as in (A); (G) Overlay of the models of MT-bound HsK5 bound to GSK-1 or AMPPNP, viewed as in panel A and at 90º compared to Figure 2B; the GSK-1-bound conformation of HsK5 is shown in mid-blue/coloured ribbon, while the AMPPNP-bound HsK5 conformation is shown in light blue; GSK-1/AMPPNP have been removed for clarity; (H) At inclusive density thresholds, connectivity between loop2 and α-tubulin is seen in HsK5-AMPPNP reconstruction (arrow), although it was not possible to model the structure of this loop, consistent with the flexibility of this connection.

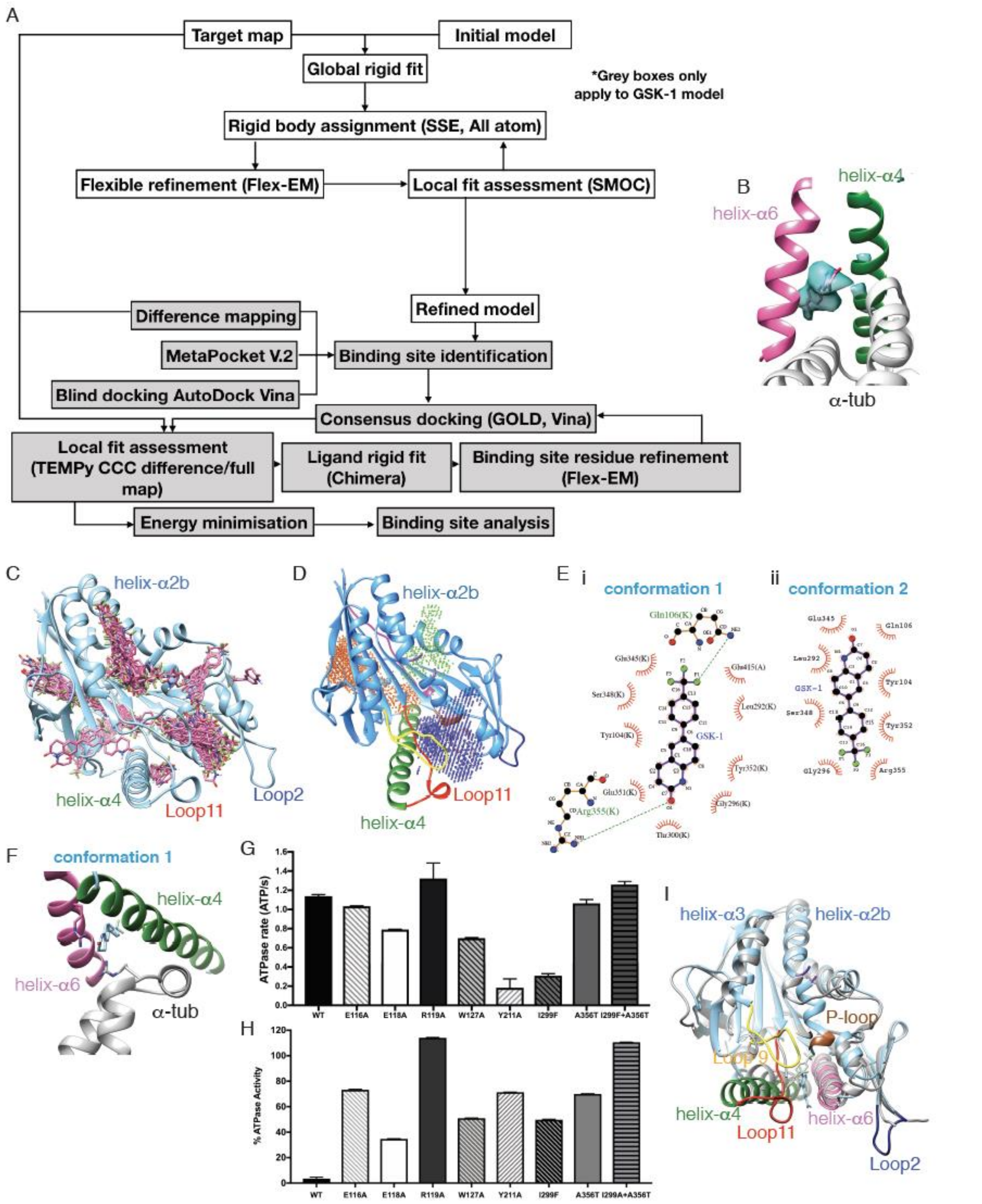

Figure S3 Related to Figure 3. Evaluation of the GSK-1 binding site to MT-bound HsK5. (A) Protocol for computationally identifying and characterising the GSK-1 binding site on MT-bound GSK-1; (B) Calculated map vs protein model difference (in turquoise) identifies major density corresponding to bound GSK-1, in the helix-α4/helix-α6; (C) Results of blind docking with AutoDock Vina (after removal of redundant poses < 2 Å) in the absence of MTs. The refined HsK5 model is shown in blue and unique GSK-1 conformations are in pink. Several discrete clusters of GSK-1 binding can be seen - their distribution is less clustered at the helix-α4/helix-α6 pocket in the absence of tubulin compared to the presence (Figure 3B); (D)

The top 3 clusters predicted by Meta-pocket 2.0 (purple, green, and, orange) shown in relation to the refined GSK-1 bound HsK5 model (blue). SSEs are also highlighted (helix-α4, green; P-loop, brown; loop11, red); (E) 2D representations generated LigPlot of the drug pocket for GSK-1 (i) conformation 1 and (ii) conformation 2; individual atoms and interactions residues are shown (C: black; O: red; N:blue; F:green); binding site residues that interact with GSK-1 via hydrophobic interactions (red semi-circles) are also indicated; (F) Depiction of the contribution of α-tubulin-Arg402 to GSK-1 binding by HsK5 as predicted by PLIP; (G) Effect of point mutagenesis on uninhibited HsK5 ATPase activity - bar graphs show mathematical mean with S.D. indicated by bars, n= 3 or 4 for each condition; (H) Sensitivity of the MT-stimulated ATPase activity of these HsK5 mutants to inhibition by 50 nM GSK-1, expressed as a % of the uninhibited activity, n= 3 or 4 for each condition; (I) Overlay of our HsK5-GSK-1-MT structure (colored as in previous figures, but depicted without tubulin) with the PVZB119-bound HsK5 structure (PDB: 3WPN, in grey (Yokoyama et al., 2015), highlighting the similarity of the drug binding sites (shown in stick representation) but also showing the extent of secondary structure disorder in HsK5-PVZB119 around the nucleotide binding site in the absence of MT binding.
